# Dietary fiber and fatty acids may impact clinical outcomes in pediatric obesity-associated asthma: insights from the SOAP study

**DOI:** 10.3389/fnut.2025.1687082

**Published:** 2025-11-03

**Authors:** Dena Samir Al-Dasooqi, Harshita Shailesh, Mohamed Nadhir Djekidel, Shaikha Alabduljabbar, Salma Hayder Ahmed, Yasmin Olabi, Nour Shallouf, Yahya Hani, Ibrahim Janahi, Annalisa Terranegra

**Affiliations:** ^1^Translational Medicine Department, Sidra Medicine, Doha, Qatar; ^2^Division of Pulmonology, Department of Pediatric Medicine, Sidra Medicine, Doha, Qatar; ^3^Department of Pediatrics, Weill Cornell Medicine – Qatar, Doha, Qatar

**Keywords:** asthma, obesity, pulmonary function, inflammation, diet, fatty acids, fiber

## Abstract

**Background and aims:**

Evidence suggests that diet influences the pathophysiology of asthma, but its role in pediatric obesity-related asthma is unclear. This case–control study aimed to explore the relationships between nutrient intake and the pathophysiology of asthma in children who are overweight or obese.

**Methods:**

Participants of the Sphingolipids in Childhood Asthma and Obesity study (100 children aged 6–17 years) were divided into four groups: normal weight with asthma (NW-A, *n* = 16); overweight or obese with asthma (OO-A, *n* = 26); normal weight (NW, *n* = 33); overweight or obese (OO, *n* = 25). Dietary intake was recorded via 3-day food diaries. Diet quantity and quality were assessed using UK Government Dietary Recommendations and dietary inflammatory index (DII) scores. Nutrient intake was compared across groups, and regression analyses were applied to identify the top contributors to asthma and obesity-associated asthma. Correlation analyses were used to assess the associations between the most important nutrients and clinical parameters.

**Results:**

Fatty acids (FAs), including saturated, monounsaturated, and polyunsaturated FAs, were identified as the most significant contributors to asthma and obesity-associated asthma, followed by several vitamins, fibers, and sugars. The relationships between nutrients and clinical parameters showed different patterns in the NW-A and OO-A groups. In NW-A, intakes of saturated, monounsaturated, and polyunsaturated FAs, including *α*-linolenic acid (PUFA 18:3, n-3), were positively associated with vital capacity and total lung capacity and inversely related to plasma levels of interleukin (IL)-10; while soluble fiber intake was negatively correlated with lung clearance index. In OO-A, FAs, including linoleic acid (PUFA 18:2, n-6) and *α*-linolenic acid, and vitamin E isoforms were positively associated with vital capacity, total lung capacity, inspiratory capacity, and forced vital capacity, and negatively associated with lung clearance index and forced expiratory volume in 1 s. Multiple saturated FA intakes were negatively associated with levels of IL-10, IL-17A, and IL-2.

**Conclusion:**

This study suggests that certain dietary components, such as FAs and fiber, may have different effects on asthma in overweight or obese children compared to normal weight children. Thus, tailored dietary modifications, guided by body mass index, could improve asthma symptoms. Randomized controlled trials are needed to confirm these associations and guide dietary recommendations.

## 1 Introduction

Asthma and obesity are among the most prevalent chronic diseases affecting children and adolescents worldwide. In the State of Qatar, asthma affects 35% of school-aged children (1), nearly half of whom are classified as overweight or obese (2).

Excess weight and obesity significantly elevate the risk of developing asthma in childhood (3, 4); meta-analyses studying this association have reported a 20% increase in asthma risk in overweight children (5) and up to a two-fold increase in those with obesity (6). Obesity is also a disease modifier of asthma (7) and has been linked to reduced asthma control, an elevated risk of exacerbations (8), increased severity and morbidity (4), and a lower quality of life (7). The relationship between asthma and obesity is not fully understood but may involve mechanisms such as obesity-mediated inflammation, the immunomodulatory effects of obesity, gene regulation, metabolic dysregulation, a sedentary lifestyle, and macro- and micronutrient intakes (6, 9).

Diet plays a critical role in the prevention, development, and management of obesity. The rise in childhood obesity is closely linked to the increasing prevalence of Western dietary patterns (10), which are characterized by the high consumption of processed and energy-dense foods, saturated fat, and sugar and the low consumption of fruits, vegetables, and whole grains. These types of diets promote excessive caloric intake and the suboptimal intake of vital nutrients.

Nutrition has been suggested to modulate the risk, severity, and presentation of allergic diseases including asthma, eczema, and rhinitis (11–23). Indeed, nutrients such as vitamin D, n-3 polyunsaturated fatty acids (PUFAs), and antioxidants have anti-inflammatory and immunoregulatory effects in asthma (24–26). Moreover, evidence suggests that adherence to dietary patterns, such as the Mediterranean diet, may reduce the risk of asthma and its associated symptoms in children (4, 27, 28). Conversely, diets high in saturated fat, such as Western diets, have been suggested to increase the risk and severity of asthma (24, 28, 29). Imbalances in macronutrient intake have also been suggested to negatively impact asthma severity (24, 30–32).

Despite these advances, the role of nutrition in pediatric obesity-related asthma remains poorly understood. We thus conducted this study to explore the relationships between dietary intake and the pathophysiological features of obesity-related asthma in children in Qatar. Specifically, we focused on identifying dietary components associated with pulmonary function and inflammatory biomarkers in children with asthma, stratified by BMI, and on determining whether the influence of specific nutrients on asthma outcomes differs by obesity status. This study was conducted as part of the Sphingolipids in Childhood Asthma and Obesity (SOAP) project, a cross-sectional study investigating the role of altered sphingolipid metabolism in children with asthma and obesity (33).

## 2 Methods

### 2.1 Study population and clinical parameters

This study included 100 children, aged 6 to 17 years, who were selected from the SOAP study cohort based on the adequacy and availability of dietary intake data. Details regarding the characteristics of the SOAP cohort, original sample size, inclusion and exclusion criteria, recruitment procedures, and data collection methods can be found in the SOAP study protocol (33). The study was approved by the Sidra Medicine IRB committee (IRB No. 1500770, 7 October 2020), and all participants and their parents provided their written consent for data to be included in the study. Participants were allocated to one of four groups based on the presence of asthma and their body mass index (BMI) z-score for age and sex: normal weight with asthma group (NW-A), overweight or obesity with asthma group (OO-A), normal weight group (NW), and overweight or obesity group (OO). According to the WHO growth standards for children aged 5–19 years, overweight was defined as a BMI-for-age z-score greater than +1 standard deviation (SD), and obesity was defined as a z-score greater than +2 SD, adjusted for sex. The methods used for diagnosing asthma are described in the SOAP study protocol (33). A variety of clinical and anthropometric data were included in our analyses: BMI; fat mass; fat-free mass; total body water; basal metabolic rate; circumferences of the hip, waist, neck, and chest; allergies; medical conditions; serum levels of albumin, thyroid stimulating hormone, free thyroxine (free T4), vitamin D, C-peptide, CO_2_, and cytokines; full blood count; lipid profile; heart rate; respiratory rate; and lung function parameters. Data on plasma cytokine levels, including IL-2, IL-5, IL-10, IL-13, IL-17A, IL-22, IL-33, IFN-*γ*, TNF-*α*, and leptin, were obtained from a previous study (34).

Pulmonary function was assessed using spirometry parameters, including forced vital capacity (FVC), forced expiratory volume in 1 s (FEV1), FEV1/FVC ratio, and forced mid-expiratory flow (FEF 25–75%), and plethysmography parameters, including airway resistance (Raw), specific airway resistance (sRaw), vital capacity (VC), inspiratory capacity (IC), functional residual capacity by plethysmography (FRCpleth), expiratory reserve volume (ERV), total lung capacity (TLC), residual volume (RV), and RV/TLC ratio. Fractional exhaled nitric oxide (FeNO) and lung clearance index (LCI) were also measured. Spirometry and plethysmography parameters were expressed as percentages of the predicted values.

### 2.2 Dietary assessment

The participants’ dietary intake was captured using 3-day food diaries, completed either by the participants themselves or by their guardians, depending on the participant’s age. Dietary data were input into Nutritionist Pro™ nutrition analysis software (Axxya Systems), and the 3-day diets were comprehensively analyzed for total energy and macro- and micronutrient contents, which were then averaged across the 3 days. The dietary inflammatory index (DII) was calculated based on each participant’s average daily nutrient intake to evaluate the quality of their diet (35, 36). Participants’ average nutrient intake was also evaluated against the UK Government Dietary Recommendations for energy, macronutrients, and fiber (37).

### 2.3 Statistical analyses

Normality of the data was tested using the Shapiro–Wilk test. Normally distributed clinical variables and nutrients were compared across the four groups using one-way analysis of variance (ANOVA), followed by Tukey’s honestly significant difference test for multiple comparisons. Clinical variables and nutrients that did not follow a normal distribution were compared across the groups via the Kruskal–Wallis test, followed by Dunn’s test for multiple comparisons with adjustments of *p*-values by Bonferroni correction. Categorical clinical variables were compared across the four groups via the chi-square test or Fisher’s exact test. The Mann–Whitney U test and the t-test were used to compare the intake of key nutrients between asthmatic groups. Ridge regression was used to identify the top nutrients associated with each phenotype. Nutrients with a Ridge coefficient larger than Q25 of the coefficients and an AUC > 0.5 were selected for correlation analyses. All variables were rank transformed to normalize the data before performing Pearson’s partial correlation analysis of nutrient intake levels and clinical variables, while controlling for sex. A two-sided *p*-value < 0.05 indicated statistical significance. Statistical analyses were performed using IBM SPSS v. 29, and GraphPad Prism 10.2.3. R version 4.4.0 was used for the Ridge regression (glmnet v4.1.8) and for plotting (ComplexHeatmap v2.18.0, ggplot2 v3.5.0).

## 3 Results

### 3.1 Study population and clinical characteristics

A sub-cohort from the SOAP study was selected for analysis based on the availability of dietary data. A total of 100 children were divided into four groups: NW-A (*n* = 16); OO-A (*n* = 26); NW (*n* = 33); and OO (*n* = 25). Demographic and clinical variables were compared across the four groups (Table 1). The analysis revealed a significantly higher percentage of males in NW-A and OO-A than OO. In accordance with the SOAP study inclusion criteria (17), BMI z-scores were significantly higher in OO-A and OO than in NW-A and NW. Among the pulmonary function parameters, the FEV1/FVC and RV/TLC ratios were lower in OO-A, and the sRaw was higher in NW-A. As expected, rhinitis, eczema, and other allergies were more prevalent in NW-A and OO-A than the control groups. Cardiometabolic parameters differed between normal weight and overweight or obese participants, with significantly higher systolic blood pressure and heart rate measured in OO-A and OO, and a significantly higher respiratory rate in NW-A (Table 1).

**Table 1:** Demographic and clinical characteristics of the study participants.

Variables	NW-A (*n* = 16)	OO-A (*n* = 26)	NW (*n* = 33)	OO (*n* = 25)	*p-*value	Pairwise comparison (*p*-value)
Sex, n (%)						NW-A vs. OO (*p* = 0.033)
Female	3 (18.8%)	4 (15.4%)	14 (42.5%)	13 (52%)	0.016^1^	OO-A vs. NW (*p* = 0.025)
Male	13 (81.3%)	22 (84.6%)	19 (57.6%)	12 (48%)		OO-A vs. OO (*p* = 0.006)
Age (years)^a^	9.8 (8.5–13.4)	9.3 (7.7–12.6)	10.7 (8.56–13.7)	12.8 (9.4–14.8)	ns^#^	ns
BMI z-score ^a^	−0.36 (−1.06–0.38)	1.98 (1.4–2.33)	−0.18 (−0.99–0.36)	1.96 (1.67–2.38)	<0.001^#^	NW-A vs. OO-A (*p* = 0.000) NW-A vs. OO (*p* = 0.000) OO-A vs. NW (*p* = 0.000) NW vs. OO (*p* = 0.000)
FEV1 pp. (%)^b^	94.1 (11.5)	93.3 (11.6)	94.1 (11.5)	100.1 (17.8)	ns*	ns
FEV1/FVC^a^	92 (80–108)	92.5 (74–10)	101.5 (83–110)	99 (51–112)	0.003^#^	OO-A vs. NW (*p* = 0.003)
FEF 25–75% pp. (%)^b^	77.7 (24.7)	74.0 (21.3)	85.4 (24.2)	89.7 (27.6)	ns*	ns
Raw pp. (%)^a^	171.8 (106.3–340.0)	176.85 (83.2–299.6)	146.5 (80–214.8)	152.5 (82.6–341.6)	ns^#^	ns
sRaw pp. (%)^a^	237.9 (173.9–342.7)	189.65 (106.2–388.6)	177.2 (85.3–311.0)	172.2 (118.5–307.0)	0.046^#^	NW-A vs. NW (*p* = 0.046)
VC pp. (%) ^b^	98.9 (10.0)	97.6 (10.3)	88.4 (12.5)	101.0 (13.2)	*p* = 0.001*	OO-A vs. NW (*p* = 0.033 NW vs. OO (*p* = 0.001)
IC pp. (%) ^b^	89.4 (27.9)	96.9 (20.9)	78.6 (19.0)	88.9 (26.1)	*p* = 0.037*	OO-A vs. NW (*p* = 0.023)
TLC pp. (%) ^a^	102.6 (86.1–128.6)	98.0 (76.5–115.4)	89.5 (71.0–133.6)	94.3 (78.4–135.0)	ns^#^	ns
RV/TLC ^b^	107.0 (25.4)	96.1 (21.3)	114.2 (21.8)	80.0 (23.9)	<0.001*	NW-A vs. OO (*p* = 0.009) OO-A vs. NW (*p* = 0.025) NW vs. OO (*p* < 0.001)
FeNO (ppb)^a^	34.6 (5–181.9)	28.6 (5.8–174.5)	17.3 (3.0–164.0)	21.6 (6.5–172.5)	ns^#^	ns
LCI ^a^	6.6 (5.4–9.0)	6.9 (6.1–9.2)	6.5 (5.5–9.2)	6.7 (5.4–8.0)	ns^#^	ns
Eczema, n (%)	7 (43.8%)	5 (19.2%)	0	2 (8%)	<0.001^2^	NW-A vs. NW (*p* < 0.001) NW-A vs. OO (*p* = 0.018) OO-A vs. NW (*p* = 0.013)
Allergies, n (%)	7 (43.8%)	4 (15.4%)	1 (3%)	2 (8%)	0.001^2^	NW-A vs. NW (*p* < 0.001) NW-A vs. OO (*p* = 0.017)
Rhinitis, n (%)	5 (31.3%)	13 (50%)	1 (3%)	0	<0.001^2^	NW-A vs. NW (*p* = 0.011) NW-A vs. OO (*p* = 0.006) OO-A vs. NW (*p* < 0.001) OO-A vs. OO (*p* < 0.001)
Systolic blood pressure (mm Hg)^a^	109.5 (96.1–114)	112.8 (106.6–118.6)	100.7 (98.7–106.3)	113 (105.3–120)	<0.001^#^	OO-A vs. NW (*p* = 0.001) NW vs. OO (*p* < 0.001)
Diastolic blood pressure (mm Hg)^b^	66.2 (6.74)	70.2 (6.22)	66.9 (6.06)	71 (7.22)	0.031*	ns
Heart rate (bpm)^a^	79.3 (72.9–85.3)	89.2 (81.7–102.9)	83 (74.0–90.5)	90.3 (83.3–101)	0.003^#^	NW-A vs. OO-A (*p* = 0.021) NW-A vs. OO (*p* = 0.047)
Respiratory rate (breaths/min)^a^	24.7 (20.2–29.2)	19.5 (18.6–26.2)	19.7 (18–22.2)	20 (18–25)	0.009^#^	NW-A vs. NW (*p* = 0.005)

^a^Median (IQR), ^b^Mean (SD). Continuous variables were compared across groups via ANOVA (*) or Kruskal–Wallis test (^#^), followed by Tukey’s honest significant difference or Bonferroni-corrected Dunn’s post-hoc test, respectively, according to data distribution. Categorical variables were compared via the chi-square test (^1^) or Fisher’s exact test (^2^). BMI, body mass index; bpm, beats per minute; breaths/min, breaths per minute; FeNO, fractional exhaled nitric oxide; FEV1, forced expiratory volume in 1 s; FEV1/FVC, forced expiratory volume in 1 s/forced vital capacity ratio; FVC, forced vital capacity; LCI, lung clearance index; ns, not significant; NW, normal weight; NW-A, normal weight with asthma; OO, overweight or obesity; OO-A, overweight or obesity with asthma; pp., percent predicted; Raw, airway resistance; RV, residual volume; sRaw, specific airway resistance; TLC, total lung capacity.

### 3.2 Nutrient intake and diet quality differ in asthmatic children according to BMI

**Figure 1:**
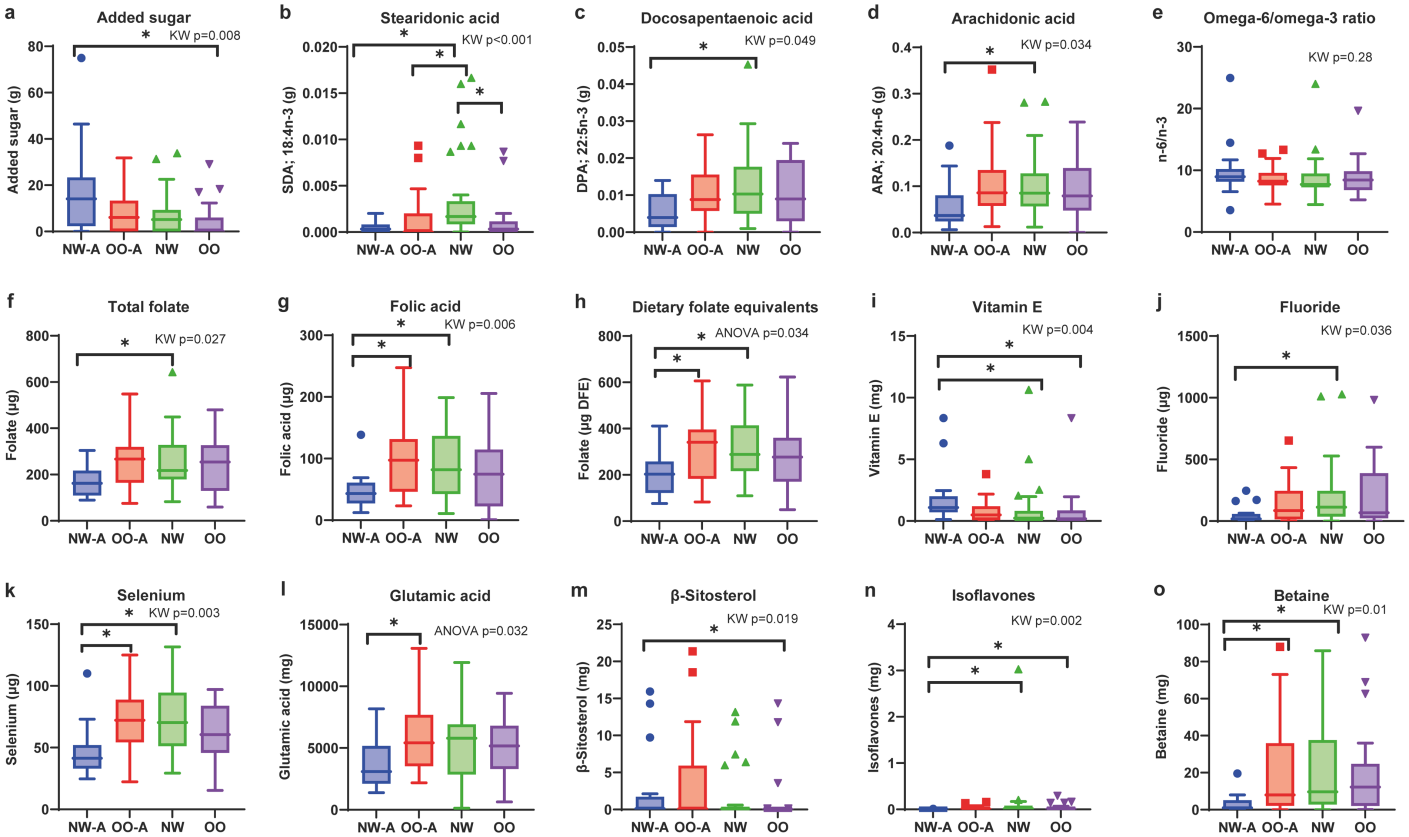
Comparisons of nutrient intakes and dietary n-6/n-3 PUFA ratio across the four groups. Box plots showing the four groups’ levels of **(a)** added sugar, **(b)** stearidonic acid, **(c)** docosapentaenoic acid, **(d)** arachidonic acid, **(e)** omega-6/omega-3 ratio, **(f)** total folate, **(g)** folic acid, **(h)** dietary folate equivalents, **(i)** vitamin E, **(j)** fluoride, **(k)** selenium, **(l)** glutamic acid, **(m)** *β*-sitosterol, **(n)** isoflavones, and **(o)** betaine. Data are presented as means and standard deviations for normally distributed nutrients **(h,l)** and medians and interquartile ranges for non-normally distributed nutrients **(a–g,i–k,m–o)**. ANOVA or the Kruskal–Wallis test was used to test differences based on data distribution, followed by Tukey’s honestly significant difference or Bonferroni-corrected Dunn’s *post-hoc* test, respectively. Only nutrients with significant overall group differences (*p* < 0.05) are shown. *p < 0.05. ARA, arachidonic acid; DFE, dietary folate equivalents; DPA, docosapentaenoic acid; NW, normal weight; NW-A, normal weight with asthma; OO, overweight or obesity; OO-A, overweight or obesity with asthma; PUFA, polyunsaturated fatty acid; SDA, stearidonic acid.

The intake of several nutrients, including added sugar, fatty acids (FAs), minerals, vitamins, and bioactive compounds, differed significantly across the four groups (Figure 1), with specific intergroup differences identified through post-hoc pairwise comparisons (Supplementary Table 1).

Added sugar intake was significantly different among the groups, with NW-A exhibiting the highest intake (Figure 1a). Among the FAs, the intakes of stearidonic acid (SDA; 18:4, n-3), docosapentaenoic acid (DPA; 22:5, n-3), and arachidonic acid (ARA; 20:4, n-6) were significantly different among groups, with NW-A showing the lowest intake (Figures 1b–d). No significant differences in the n-6/n-3 PUFA ratio were observed. Although not statistically significant, NW-A presented the highest n-6/n-3 ratio, with a median of 8.96 (IQR 8.16–10.22; Figure 1e).

The intakes of total folate, folic acid, and DFE were significantly lower in NW-A, whereas vitamin E was significantly higher in NW-A (Figures 1f–h). Other micronutrients showed different intakes among the four groups, including fluoride, selenium, glutamic acid, and bioactive compounds (*β*-sitosterol, isoflavones, betaine; Figures 1i–o). The detailed results of the pairwise group comparisons are available in Supplementary Table 1.

In summary, we observed lower nutrient intake in NW-A, except for added sugar and vitamin E, whereas children in OO-A showed a dietary intake similar to their non-asthmatic counterparts.

**Figure 2:**
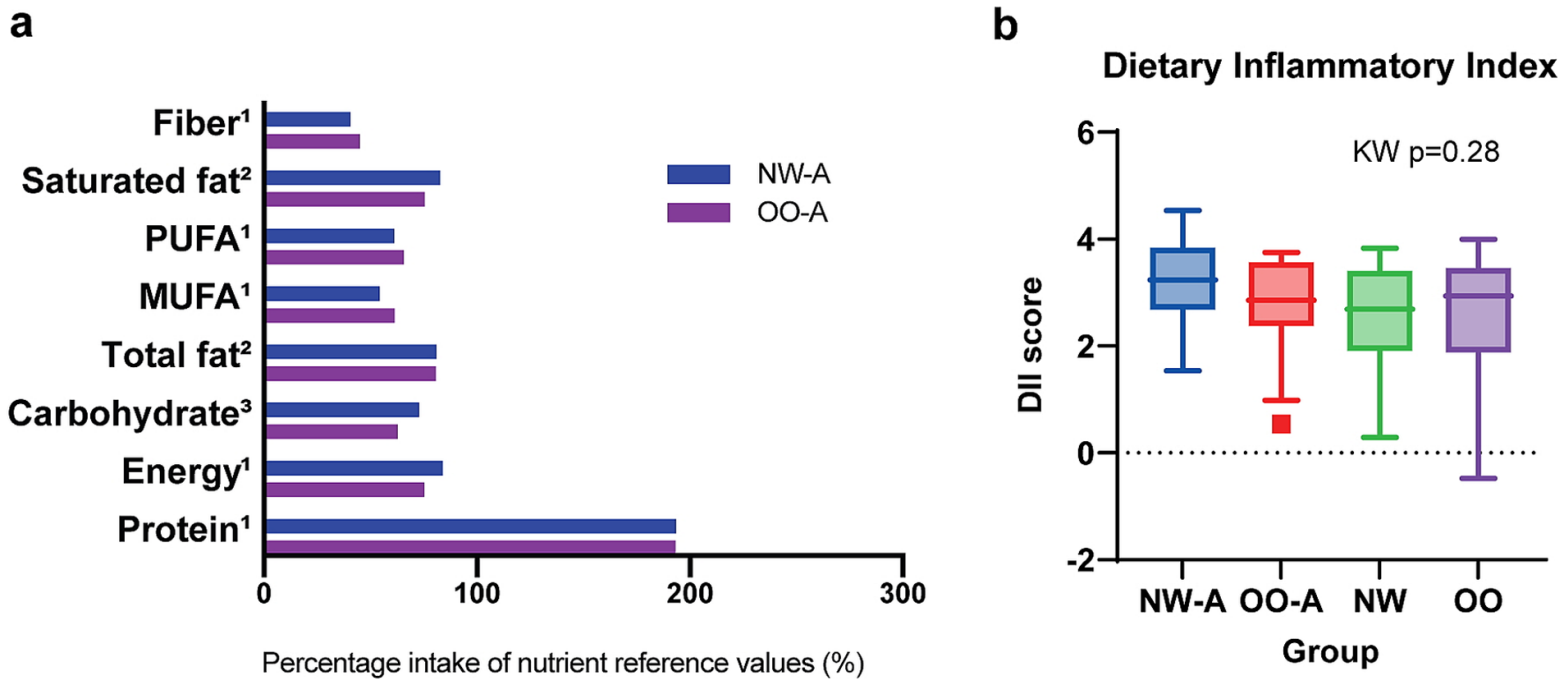
Comparison of diet quantity and quality between study groups. Panel **(a)** shows energy, macronutrient, and fiber intake in NW-A and OO-A compared to the UK Government Dietary Recommendations. Nutrient reference values represent either ^1^average, ^2^maximum, or ^3^minimum recommended intake. Groups were compared via the Mann–Whitney U test or t-test. Panel **(b)** shows the distribution of DII scores across the four groups. Data are presented as medians and interquartile ranges. Group comparisons were performed via the Kruskal–Wallis (KW) test. MUFA, monounsaturated fatty acids; NW, normal weight; NW-A, normal weight with asthma; OO, overweight or obesity; OO-A, overweight or obesity with asthma; PUFA, polyunsaturated fatty acids.

Due to the lower intake of many nutrients in NW-A compared to OO-A, we conducted a quantitative analysis to assess the adequacy of dietary intake in both groups when compared to the UK Government Dietary Recommendations for daily energy, macronutrient, and fiber intake (37). Average energy, macronutrient, and fiber intake per group were expressed as percentages of reference values and compared between NW-A and OO-A via the Mann–Whitney U test or t-test. No significant differences were found between the two groups in terms of energy or any of the nutrients. However, both groups exceeded the recommended average intake for protein by approximately 93%. Furthermore, NW-A and OO-A, respectively met only 73 and 63% of the recommended minimum carbohydrate intake and just 41 and 45% of the recommended minimum fiber intake (Figure 2a). This analysis was also performed for NW and OO and yielded very similar results to the asthmatic groups (Supplementary Figure 1). An assessment of diet quality using the DII indicated a high dietary inflammatory potential in all groups. Comparisons of average DII scores revealed no significant differences between groups; however, NW-A presented the highest DII score (Figure 2b; Supplementary Table 1). Although differences among the groups were not significant, diet quality showed negative trends in asthmatic patients.

Overall, single nutrients were consumed differently in NW-A and OO-A, with NW-A children consuming lower amounts of FAs, folates, minerals, and bioactive compounds, in contrast to their higher intake of added sugar. These differences are reflected in the poorer diet quality.

### 3.3 Single nutrients are associated with pulmonary function and clinical outcomes

**Figure 3:**
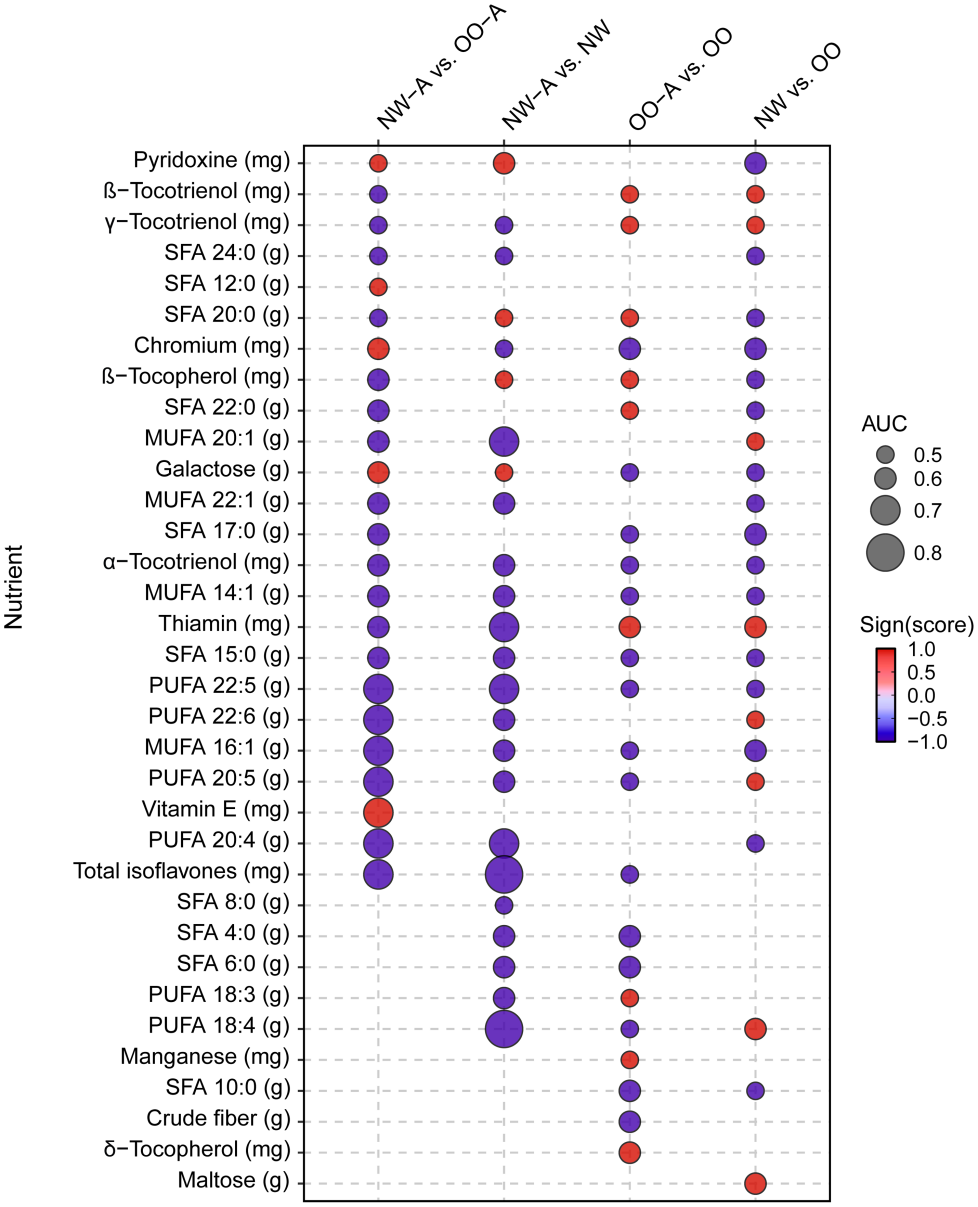
Ridge regression analysis of nutrient intake among the study groups. Ridge regression was performed on nutrient data, controlling for sex, in NW-A vs. OO-A, NW-A vs. NW, OO-A vs. OO, NW vs. OO. The image displays the most predictive nutrients among those with a coefficient larger than Q25 and an AUC > 0.5. MUFA, monounsaturated fatty acid; NW, normal weight; NW-A, normal weight with asthma; OO, overweight and obese; OO-A, overweight or obese with asthma; PUFA, polyunsaturated fatty acid; SFA, saturated fatty acid.

To investigate the impact of diet quality and single nutrient intake on asthma, we next conducted correlation analyses to investigate the associations between various measures of pulmonary function and nutrient intake in the study groups. To limit the collinearity effect between the measured 125 nutrients, we first ran a Ridge regression to identify nutrients predictive of NW-A vs. OO-A, NW-A vs. NW, OO-A vs. OO or NW vs. OO, and used them for the following correlation analyses. We selected the top best-performing nutrients with coefficients larger than the 25th percentile (Q25) and an AUC > 0.5, as shown in Figure 3. A full list of the significant nutrients is available in Supplementary Table 2. Among them, the SDA (PUFA 18:4) and total isoflavones reached the highest AUC (0.84 and 0.88, respectively) and exhibited a stronger negative association with asthma in NW-A compared to NW (Figure 3). ARA (PUFA 20:4, n-3), eicosapentaenoic acid (EPA; PUFA 20:5, n-6), docosahexaenoic acid (DHA; PUFA 22:6, n-3), DPA (PUFA 22:5), palmitoleic acid (MUFA 16:1), and total isoflavones showed the strongest negative predictive scores and highest AUCs (>0.70) for obesity-associated asthma (NW-A vs. OO-A). Conversely, vitamin E was the strongest positive predictor (AUC = 0.73). Comparisons of OO-A vs. OO and NW vs. OO yielded weaker associations overall, with AUCs lower than 0.60 for all nutrients (Figure 3).

**Figure 4:**
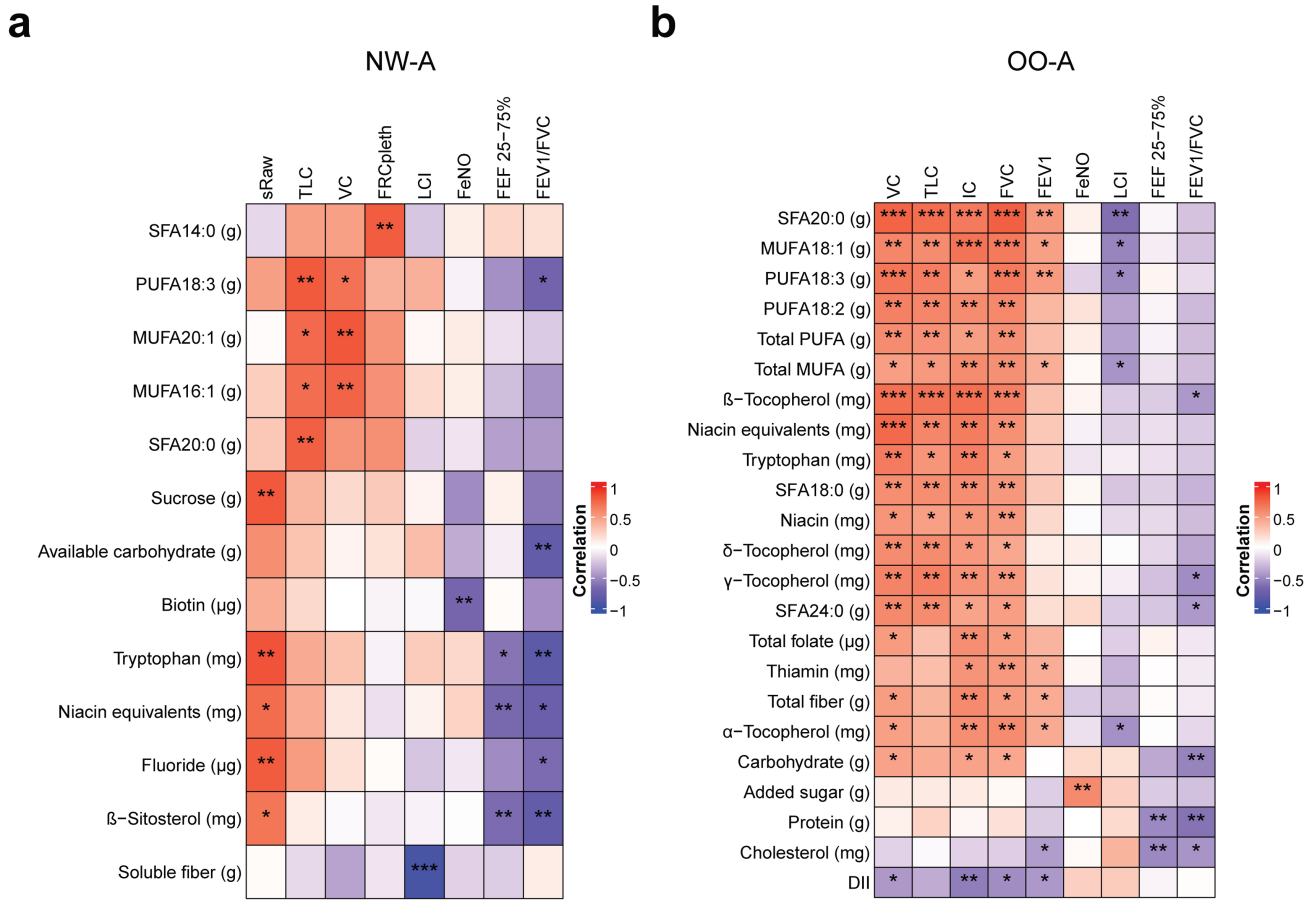
Correlations between nutrient intake and pulmonary function parameters. Pearson’s partial correlation was performed on ranked data, controlling for sex, in NW-A **(a)** and OO-A **(b)**. *p < 0.05, **p < 0.01, ***p < 0.001. DII, dietary inflammatory index; FEF 25–75%, forced mid-expiratory flow; FeNO, fractional exhaled nitric oxide; FEV1, forced expiratory volume in 1 s; FEV1/FVC, forced expiratory volume in 1 s/forced vital capacity ratio; FRCpleth, functional residual capacity by plethysmography; FVC, forced vital capacity; IC, inspiratory capacity; LCI, lung clearance index; MUFA, monounsaturated fatty acids; NW-A, normal weight with asthma; OO-A, overweight or obesity with asthma; PUFA, polyunsaturated fatty acids; SFA, saturated fatty acid; sRaw, specific airway resistance; TLC, total lung capacity; VC, vital capacity.

We then performed correlation analyses between the selected nutrients from the regression analysis and the pulmonary function and other clinical parameters, separately. In NW-A, significant positive correlations were observed between VC and TLC and a range of SFAs, MUFAs, and PUFAs, including the *α*-linolenic acid (ALA; 18:3, n-3). FEF 25–75% and the FEV1/FVC ratio showed strong negative correlations with ALA, available carbohydrates, tryptophan, niacin equivalents, and *β*-sitosterol, while sRaw showed positive correlations with these nutrients and sucrose. LCI was strongly negatively correlated with soluble fiber and FeNO with biotin (Figure 4a). The full results can be found in Supplementary Table 3.

In OO-A, significant positive correlations were seen between VC, TLC, IC, FVC, FEV1, and many SFAs, MUFAs, and PUFAs, including arachidic acid (SFA 18:2) and ALA. VC, TLC, IC, and FVC were also positively correlated with niacin equivalents, tryptophan, and vitamin E isoforms, including *α*-, *β*-, *γ*-, and *δ*-tocopherol. VC, IC, and FVC were positively correlated with total folate, total dietary fiber, and carbohydrates. Negative correlations were observed between LCI and arachidic acid (SFA 20:0), oleic acid (MUFA 18:1), total MUFAs, ALA (PUFA 18:3, n-3), and vitamin E (*α*-tocopherol). The FEF 25–75% and FEV1/FVC ratio were negatively correlated with protein, carbohydrate, cholesterol intake, and vitamin E (*β*- and *γ*-tocopherol, Figure 4b). The full results can be found in Supplementary Table 4. The OO-A group showed a higher number of associations between FA consumption and pulmonary function, indicating dietary fats have a stronger impact in obese asthmatic patients.

**Figure 5:**
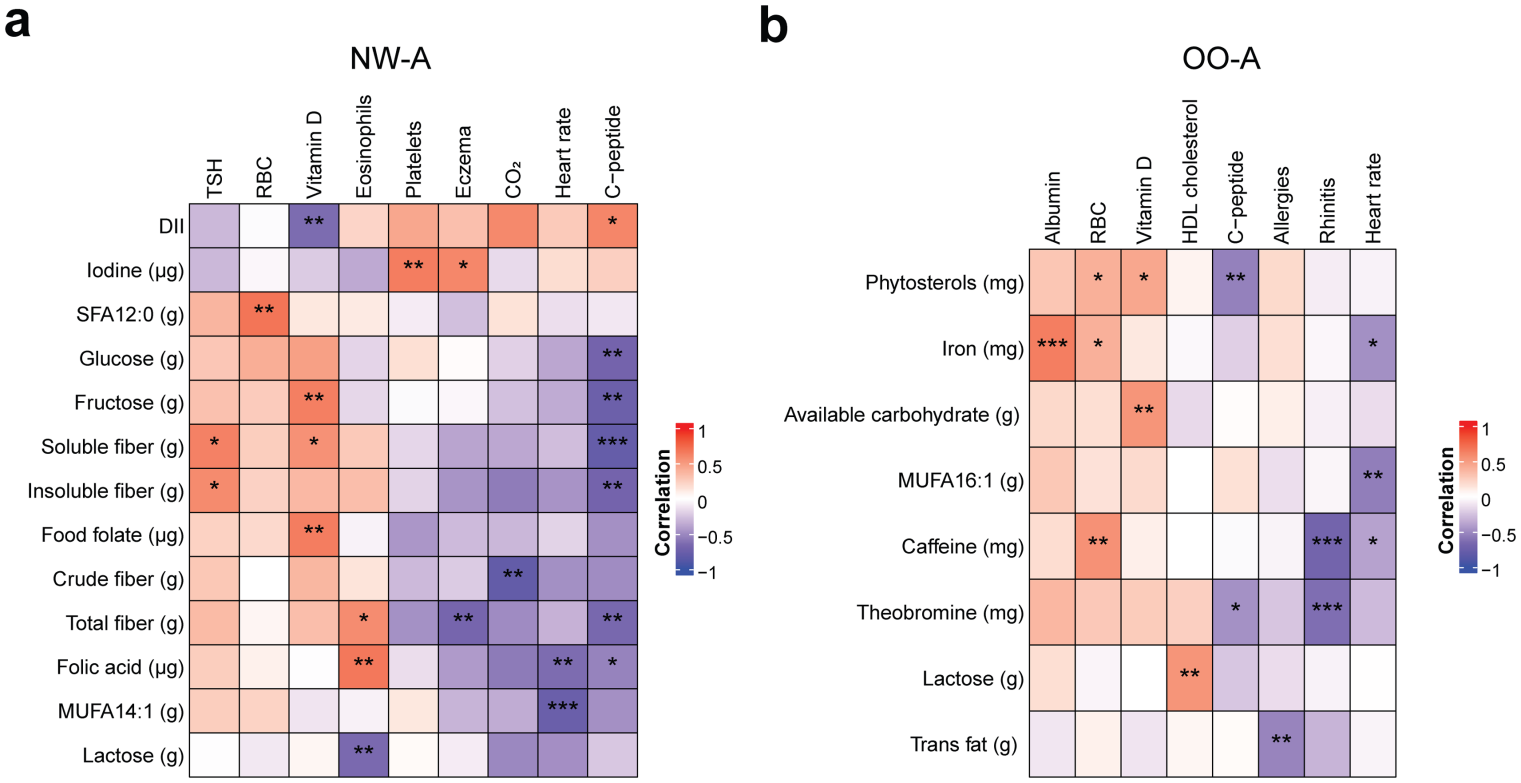
Correlations between nutrient intake and clinical outcomes. Pearson’s partial correlation was performed on ranked data, controlling for sex, in NW-A **(a)** and OO-A **(b)**. *p < 0.05, **p < 0.01, ***p < 0.001. DII, dietary inflammatory index; HDL, high density lipoprotein; MUFA, monounsaturated fatty acid; NW-A, normal weight with asthma; OO-A, overweight or obesity with asthma; RBC, red blood cell; SFA, saturated fatty acid; TSH, thyroid stimulating hormone.

To further explore the associations between nutrient intake and other clinical outcomes in children with asthma, we conducted additional correlation analyses of both NW-A and OO-A data. The analyses yielded different results in each group, with several nutrients showing correlations with C-peptide, serum vitamin D, and rhinitis. In NW-A specifically, high C-peptide, eosinophils, and serum vitamin levels were the most impactful. Serum vitamin D levels were positively correlated with fructose, food folate, and soluble fiber. Eosinophils were negatively correlated with lactose and positively with folic acid and total dietary fiber. High C-peptide was associated with a lower intake of glucose, fructose, fibers, and folic acid (Figure 5a; Supplementary Table 5). Fiber intake was associated with most of the clinical parameters in NW-A.

In OO-A, the incidence of rhinitis showed the strongest negative associations with the intake of theobromine and caffeine. Heart rate was also negatively correlated with caffeine, MUFA 16:1, and iron, while C-peptide levels were negatively correlated with phytosterols and theobromine. Albumin showed the highest positive correlation with iron intake. Red blood cells were positively associated with the intake of iron, phytosterols, and caffeine. Serum vitamin D levels were positively correlated with phytosterol intake and available carbohydrates, and HDL cholesterol was positively correlated with lactose consumption (Figure 5b; Supplementary Table 6). In OO-A, associations between nutrients and clinical parameters were generally isolated, occurring with single parameters rather than showing consistent links across multiple parameters.

In summary, both pulmonary function and clinical outcomes showed strong correlations with single nutrients, mainly fatty acids, folic acid, sugars, and fibers, with a higher number of associations seen in the NW-A group.

### 3.4 Single nutrients are associated with inflammatory cytokines

Because proinflammatory diets were consumed by all groups, we analyzed nutrient intake in relation to plasma inflammatory cytokine levels to investigate the potential downstream effects. Firstly, when cytokine levels were compared across groups, significant differences in IL-5, IL-13, IL-33, TNF-*α*, and leptin levels were revealed. NW-A, OO-A, and OO had the highest levels of all these cytokines, except leptin, although they were significantly higher in the obese groups only (Supplementary Table 7).

**Figure 6:**
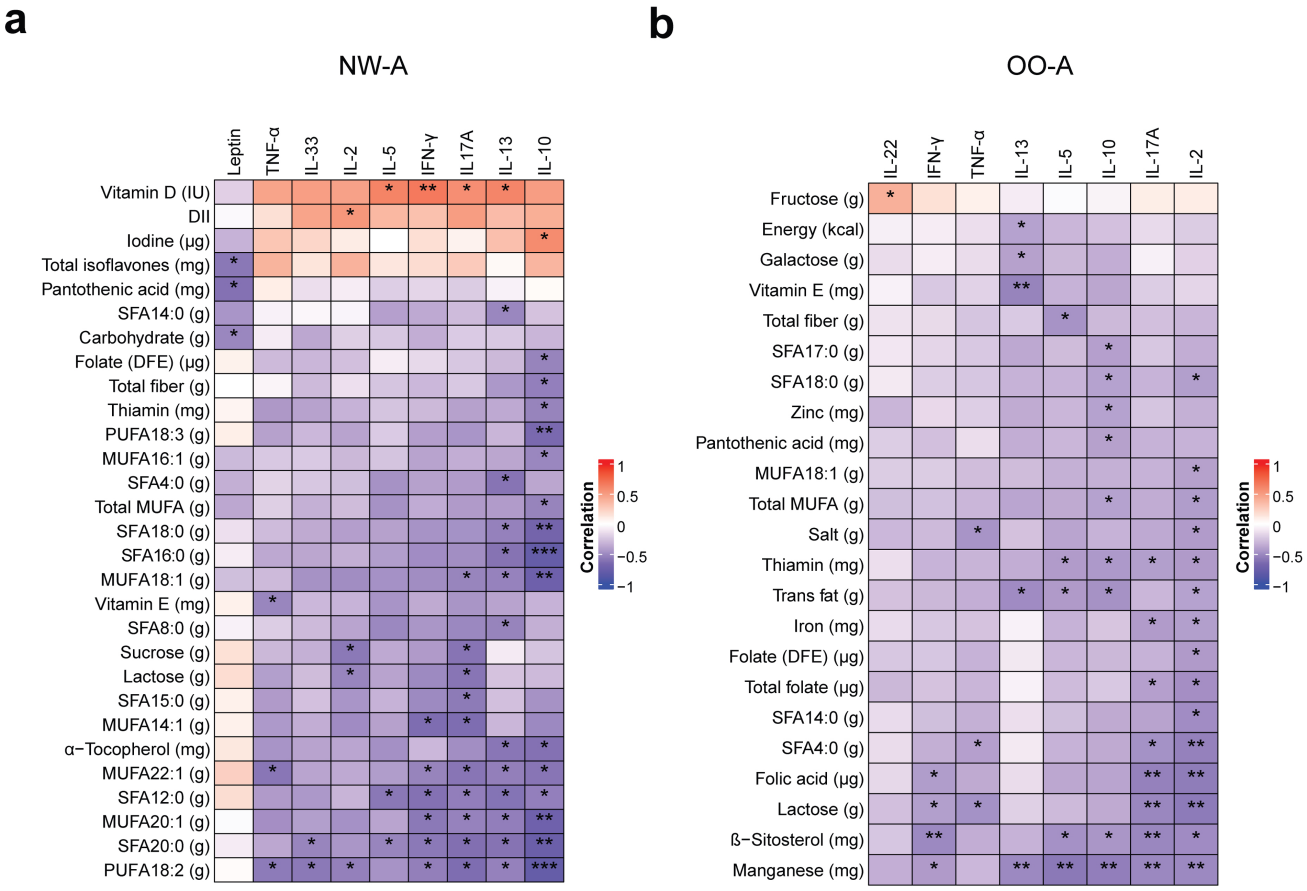
Correlations between nutrient intake and plasma cytokine levels. Pearson’s partial correlation was performed on ranked data, controlling for sex, in NW-A **(a)** and OO-A **(b)**. *p < 0.05, **p < 0.01, ***p < 0.001. DII, dietary inflammatory index; DFE, dietary folate equivalents; IFN, interferon; IL, interleukin; MUFA, monounsaturated fatty acids; NW-A, normal weight with asthma; OO-A, overweight or obesity with asthma; PUFA, polyunsaturated fatty acids; SFA, saturated fatty acid; TNF, tumor necrosis factor.

We then performed correlation analysis to identify the single nutrients associated with variations in plasma cytokine levels (Figure 6). In NW-A, IL-10, IL-13, IL-17A, and IFN-*γ* showed multiple significant correlations with individual nutrients, the majority of which were FAs. IL-10 demonstrated the strongest correlations and was negatively correlated with several nutrients, including dietary fiber, folate (DFE), thiamin, *α*-tocopherol, total MUFAs, and a range of individual FAs: hexadecenoic acid (MUFA 16:1), oleic acid (MUFA 18:1), gondoic acid (MUFA 20:1), erucic acid (MUFA 22:1), LA (PUFA 18:2, n-6), ALA (PUFA 18:3, n-3), lauric acid (SFA 12:0), palmitic acid (SFA 16:0), stearic acid (SFA 18:0), and arachidic acid (SFA 20:0). Conversely, iodine intake was positively correlated with IL-10 levels. Levels of IL-13 were negatively correlated with several individual SFAs and MUFAs, LA, and *α*-tocopherol, but positively correlated with vitamin D intake. Similarly, IL-17A showed a positive correlation with vitamin D intake and negative correlations with multiple SFAs and MUFAs, total PUFAs, and LA. IFN-*γ* was positively correlated with vitamin D intake and negatively correlated with a smaller subset of FAs than the other cytokines. TNF-*α* levels were negatively correlated with vitamin E, LA, and MUFA 22:1 intake. IL-33, IL-2, and IL-5 showed the lowest number of associations (Figure 6a). The full set of results can be found in Supplementary Table 8.

In OO-A, several nutrients showed negative correlations with both pro- and anti-inflammatory cytokines, with the strongest and most abundant correlations observed with IL-2, IL-17A, IL-10, and IL-13. IL-2 showed strong negative correlations with manganese, lactose, folates, iron, total MUFAs, trans FAs, salt, thiamin, and multiple SFAs, including butyric acid (SFA 4:0), myristic acid (SFA 14:0), and stearic acid (SFA 18:0). IL-17A was negatively correlated with manganese, *β*-sitosterol, lactose, folic acid, total folate, thiamin, iron, and butyric acid (SFA 4:0). Unlike in NW-A, IL-10 in OO-A was negatively correlated with manganese, *β*-sitosterol, thiamin, pantothenic acid (vitamin B5), zinc, total trans FAs and saturated fats, heptadecanoic acid (SFA 17:0), and stearic acid (SFA 18:0). IL-13 showed strong negative correlations with total energy, total FAs, vitamin E, galactose, and manganese (Figure 6b). The remaining cytokines showed a limited number of associations. The full results can be found in Supplementary Table 9.

Inflammatory cytokine levels, like pulmonary function and clinical parameters, were mainly correlated with the intake of various fatty acids and folates. The data indicate that different cytokines were associated with specific types of fatty acids, mainly MUFAs in NW-A and SFAs in OO-A.

**Figure 7:**
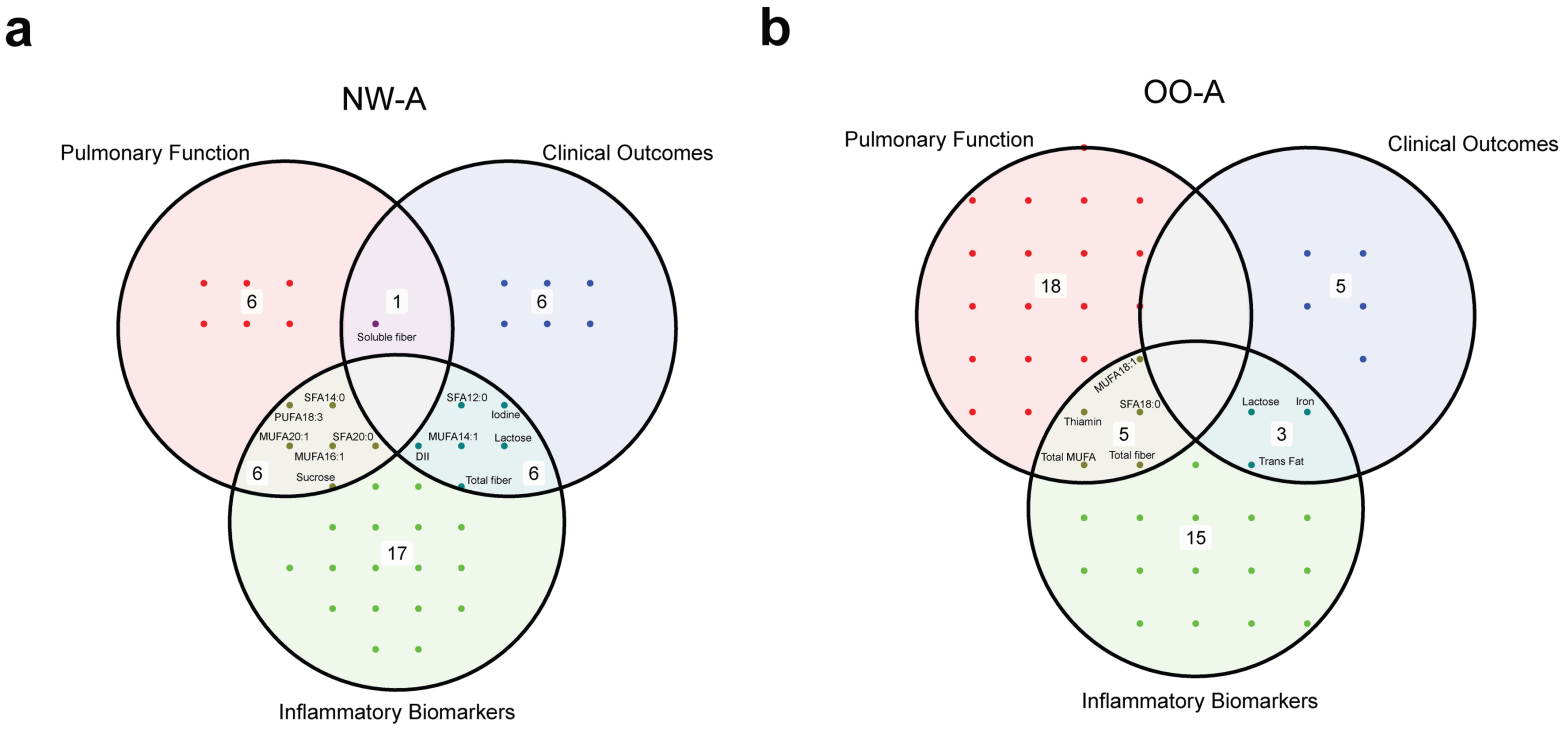
Venn diagrams showing the overlap of significant nutrients identified in the correlation analyses with pulmonary function parameters, clinical outcomes, and inflammatory biomarkers, in NW-A **(a)** and OO-A **(b)**. Labels within the circles indicate common elements such as ‘sucrose’, ‘soluble fiber’, and ‘trans fat’. DII, dietary inflammatory index; MUFA, monounsaturated fatty acid/s; NW-A, normal weight with asthma; OO-A, overweight or obesity with asthma; PUFA, polyunsaturated fatty acid; SFA, saturated fatty acid.

Venn diagrams were used to identify nutrients that were common between the correlation analyses with pulmonary function parameters, clinical outcomes, and inflammatory biomarkers (Figures 7a,b). The analysis indicated that fatty acids and fibers were the nutrient groups consistently present across all three analyses.

## 4 Discussion

Nutrition can influence both the risk and management of asthma by modulating inflammatory and immune responses (4, 24–29). However, there is insufficient evidence to support specific dietary recommendations for pediatric asthmatic populations while considering the co-occurrence of obesity. Our study showed significant differences in the intake of various nutrients across the study groups, including PUFAs, added sugar, vitamins, minerals, and bioactive compounds. NW-A consistently exhibited the lowest intake of these nutrients, except for vitamin E and added sugar, which were highest in this group. Several nutrients, including PUFAs, folic acid, and bioactive compounds, have been previously linked to beneficial effects on asthma risk and symptoms (15, 38–42). Beyond respiratory outcomes, PUFAs are widely recognized for their anti-inflammatory, cardioprotective, and metabolic benefits (43, 44). On the other hand, added sugars have been suggested to exacerbate airway inflammation (45, 46). Our dietary quantitative analysis also revealed an excessive intake of protein and an inadequate intake of MUFAs, PUFAs, and fiber in both asthmatic groups.

The intake of FAs showed dissimilarities among our cohorts and was strongly associated with clinical parameters related to asthma and obesity. In alignment with our findings, studies have shown that patients with severe asthma tend to consume more dietary fat than their healthy counterparts (47, 48). Higher fat intake is related to increased airway responsiveness and inflammation, and poorer lung function (24, 48, 49). In a randomized controlled trial, the consumption of a high-fat meal resulted in a significant increase in plasma levels of total FAs, SFAs, MUFAs, and PUFAs, which were positively associated with levels of neutrophils and other inflammatory markers in sputum and negatively associated with FVC, FEV1, and the FEV1/FVC ratio of patients with asthma (50). Significant associations were observed between certain FAs, pulmonary function, and inflammation in the OO-A group. These findings suggest that dietary interventions specifically targeting FA intake may enhance clinical outcomes in obese patients with asthma. Additional research and clinical trials are necessary to validate this hypothesis.

To investigate the relationships between diet and inflammation in asthma, we assessed the inflammatory potential of the children’s diets and examined the associations between inflammatory biomarkers and individual nutrients. The DII analysis revealed proinflammatory dietary profiles in all groups, with NW-A showing the greatest inclination toward a proinflammatory diet, though significance was not reached. Recent studies reported a strong association between higher DII scores and the risk and burden of asthma (35, 51–54). Higher DII scores have also been associated with increased systemic inflammation and reduced lung function, including lower FVC, FEV1, mid-expiratory flow rate 25 to 75%, and PEF (35, 52, 54, 55). We found similar associations between the DII and lung function parameters, including VC, IC, FVC, and FEV1, but only in OO-A, implying that BMI is a key factor in the pathophysiology of asthma. NW-A had the highest DII score, whereas OO-A showed higher levels of leptin, an inflammatory cytokine, which is elevated in obesity (56) and plays an important role in respiratory diseases, including asthma (57). Current literature and our findings indicate that using the DII alongside conventional inflammatory biomarkers can serve as a reliable tool for assessing inflammation in patients at diagnosis. In addition, dietary improvements may help reduce symptoms, particularly in obese patients affected with asthma.

When we tested for nutrients associated with proinflammatory cytokines, FAs showed the most prominent negative associations with IL-2, IL-10, IL-13, and IL-17A, which play key roles in the inflammatory processes underlying asthma. Previous studies have shown the effects of FAs in modulating these cytokines. For instance, IL-2 promotes T cell proliferation and modulates cytokine production (58). An animal study found that a higher dietary content of SFAs, n-6 PUFAs, and n-3 PUFAs reduced IL-2 production in a dose-dependent manner, with SFAs exhibiting the weakest effects and n-3 PUFAs exhibiting the strongest effects (59). IL-13 is a key driver of Th2-mediated asthma, and it promotes airway hyperresponsiveness, mucus overproduction, and eosinophilic inflammation, making it a major contributor to airway remodeling and asthma severity (60). Docosahexaenoic acid (DHA; 22:6, n-3) has been shown to inhibit IL-13 promoter activation and IL-13 expression in mice (61). IL-17A, primarily associated with neutrophilic inflammation, is implicated in severe and steroid-resistant asthma; it enhances proinflammatory mediator production and works with IL-13 to amplify airway inflammation (60). Supplementation with the n-3 PUFAs DHA and EPA (20:5, n-3) in asthmatic children was reported to result in reduced plasma levels of IL-17A and TNF-*α* (62). In NW-A, we observed consistent negative associations between MUFA and PUFA intake and several proinflammatory cytokines. The mechanisms by which PUFAs and their metabolites influence inflammation remain unclear, though they are thought to modulate inflammatory processes by regulating signal transduction and cytokine gene expression (63, 64). Evidence has shown that metabolites of n-3 PUFAs can inhibit proinflammatory cytokine secretion by T cells (61, 65). These studies and others have indicated that individual FAs have distinct effects. We also found that MUFAs were primarily associated with cytokines in NW-A, while SFAs were linked to these markers in OO-A. Consequently, differences in dietary FAs may account for the contrasting inflammatory profiles observed between NW-A and OO-A, further supporting the role of dietary changes as a potential intervention for improving clinical outcomes in obesity-associated asthma.

We found IL-10 levels were inversely correlated with SFA, MUFA, and PUFA intake in NW-A and with trans FA intake in OO-A. IL-10, an immunosuppressive cytokine that inhibits leukocyte function and downregulates proinflammatory cytokine production (66–68), is critical for resolving inflammation in the lungs (69). IL-10 gene expression was previously reported to be reduced in people with asthma (68, 70, 71). Interestingly, a higher intake of PUFAs is believed to reduce the expression of IL-10 (63, 72). Similarly, SFAs have been shown to decrease IL-10 production in murine adipocytes (73). Dietary FAs act through diverse biological pathways and may affect IL-10 levels in complex and nuanced ways. Rather than indicating a direct suppressive effect on IL-10, the inverse associations observed between IL-10 levels and the intake of unsaturated FAs may instead reflect a shared functional pathway. As described earlier, a higher consumption of nutrients such as n-3 PUFAs may be associated with reduced proinflammatory signaling and thereby diminished IL-10 expression.

The beneficial effects of FA intake in reducing inflammation may also contribute to improved lung function. In fact, we observed multiple positive correlations between SFA, MUFA, and PUFA intake and pulmonary function parameters in OO-A, including FVC, FEV1, VC, IC, and TLC. Similar associations were observed in NW-A but were much less pronounced. Several studies have demonstrated a relationship between FA intake and pulmonary function in asthma and chronic obstructive pulmonary disease, suggesting that dietary FAs have a key role in promoting respiratory health (74–76). A randomized controlled trial showed that supplementation with an n-3 PUFA-rich oil resulted in significantly increased FVC and FEV1 in participants with asthma, while an n-6 PUFA-rich oil did not (77). Farjadian et al. also reported improvements in the FEV1/FVC ratio and PEF in asthmatic children after supplementation with n-3 PUFAs (62). In a cross-sectional study, McKeever et al. did not find any protective effects of n-3 PUFA intake on asthma but suggested that n-6 PUFA intake could result in reduced FEV1 (75). Previous studies have also found inverse associations between the intake of n-3 PUFAs, particularly ALA, and levels of exhaled nitric oxide in patients with asthma (78). The discrepancies in these studies could have been due to the heterogeneity of the patients. As observed in our study, BMI can influence the immune response to PUFA intake. Another factor impacting n-3 PUFAs’ effects is the presence of their n-6 counterparts. Our analysis did not show significant differences in the n-6/n-3 PUFA ratio among the four groups, but it was highest in NW-A. The balance of dietary PUFAs also plays a critical role in asthma. A higher n-6/n-3 ratio is associated with increased asthma morbidity, while a lower ratio may have protective effects (24, 78–81). A higher intake of n-3 PUFAs may mitigate inflammatory effects. Therefore, maintaining a balanced or lower n-6/n-3 ratio, favoring n-3 intake, may help reduce asthma severity and modulate inflammatory response (79).

In addition to FAs, fiber intake was also associated with improved lung function and clinical parameters in our study. In NW-A, a very strong negative correlation was observed between soluble fiber and LCI, and different types of fibers and C-peptide. In OO-A, total fiber intake was positively correlated with FVC, VC, IC, and FEV1. Higher fiber intake has been linked to improved lung function, e.g., higher FEV1, FVC, and FEV1/FVC ratios, in patients with asthma and other respiratory diseases (48, 82, 83), and patients with severe asthma tend to consume less fiber than healthy individuals (24). In addition to these respiratory effects, dietary fiber contributes to reduced cardiometabolic risk, improved glycemic control and lower all-cause mortality (84, 85). Furthermore, inverse associations have been established between fiber intake and IL-6, TNF-*α* receptor-2, and C-reactive protein (86, 87). Dietary fiber exerts anti-inflammatory effects by altering the gut microbiota and metabolite composition (24, 88). Gut bacteria metabolize fiber into short-chain fatty acids, which have been shown to reduce airway inflammation in mice (89). Gut and lung microbiome dysbiosis has been implicated in the development and severity of asthma (90) and airway inflammation (91–93). Dietary fiber-induced alterations in gut microbiota have been shown to modulate lung immunity through the gut-lung axis (94). Controversially, we detected a negative correlation between IL-10 and total fiber intake in NW-A; this may be explained by the low fiber intake of this cohort (i.e., less than 50% of the recommended daily intake), which may be insufficient to elicit the expected beneficial effects. Nevertheless, given the numerous negative associations between IL-10 and anti-inflammatory nutrients, such as *α*-tocopherol, fiber, and certain FAs in the asthmatic groups, it is plausible that these associations arise indirectly via a general reduction in inflammatory signaling, and further research is needed.

To our knowledge, this was the first study to undertake a comprehensive analysis of more than 100 nutrients in relation to BMI and clinical outcomes in pediatric asthma. However, there are some limitations worth noting. Dietary intake was assessed using food diaries, often completed by the participants’ guardians, which could have introduced bias due to misreporting. In addition, dietary intake was assessed over 3 days, limiting our ability to capture long-term dietary patterns and raising the potential for reverse causality. Use of nutritional supplements was not queried, which may have led to an underestimation of nutrient intake. Physical activity was also not considered, despite being recognized as a potential confounding factor that may impact asthma outcomes (95). Another limitation was the combined grouping of overweight and obese participants, which may have masked or weakened obesity-specific associations between nutrition and asthma due to differing metabolic characteristics.

## 5 Conclusion

This study has highlighted the importance of diet as a modifiable factor in improving asthma outcomes. We demonstrated that normal-weight asthmatic children have a different nutrient intake profile compared with those who are overweight or obese. Key associations were identified between asthma-related clinical indicators and specific nutrients, including FAs and dietary fiber, suggesting that these nutrients have differential effects on inflammation and asthma outcomes depending on BMI. Our findings suggest that replacing dietary saturated fat with unsaturated fats, increasing fiber-rich foods, and reducing inflammatory food intake may aid asthma control. The variations in nutrient intake and clinical parameters between NW-A and OO-A reflect the intricate relationship between nutrition and asthma pathophysiology and emphasize the role of BMI in diet-disease interactions. Considering that chronic systemic inflammation is associated with obesity, interventions tailored to individual clinical profiles and BMI could be used to optimize asthma management in children. Structured randomized controlled trials are needed to evaluate the clinical efficacy of anti-inflammatory dietary interventions and to explore the complex mechanistic interplay between asthma, obesity, and nutrition. Finally, given the limitations of self-reported dietary data, biomarker-based analyses of nutrient status would strengthen the validity of the observed associations between micronutrients and clinical parameters and provide deeper insights into the mechanisms driving these relationships.

## Data Availability

The raw data supporting the conclusions of this article will be made available by the authors, without undue reservation.

## References

[1] Hammoudeh S Hani Y Alfaki M Omar N El Dimassi D Nowir K. The prevalence of asthma, allergic rhinitis, and eczema among school-aged children in Qatar: a global asthma network study. Pediatr Pulmonol. (2022) 57:1440–6. doi: 10.1002/ppul.25914, PMID: 35362672

[2] Al-Thani M Al-Thani A Alyafei S Al-Chetachi W Khalifa SE Ahmed A. The prevalence and characteristics of overweight and obesity among students in Qatar. Public Health. (2018) 160:143–9. doi: 10.1016/j.puhe.2018.03.020, PMID: 29704956

[3] Lang JE Bunnell HT Hossain MJ Wysocki T Lima JJ Finkel TH. Being overweight or obese and the development of asthma. Pediatrics. (2018) 142:e20182119. doi: 10.1542/peds.2018-2119, PMID: 30478238

[4] Averill SH Forno E. Management of the pediatric patient with asthma and obesity. Ann Allergy Asthma Immunol. (2024) 132:30–9. doi: 10.1016/j.anai.2023.10.001, PMID: 37827386 PMC10760917

[5] Hossny E Adachi Y Anastasiou E Badellino H Custovic A El-Owaidy R. Pediatric asthma comorbidities: global impact and unmet needs. World Allergy Organ J. (2024) 17:100909. doi: 10.1016/j.waojou.2024.100909, PMID: 38827329 PMC11141278

[6] Vijayakanthi N Greally JM Rastogi D. Pediatric obesity-related asthma: the role of metabolic dysregulation. Pediatrics. (2016) 137:e20150812. doi: 10.1542/peds.2015-0812, PMID: 27244776 PMC4845863

[7] Peters U Dixon AE Forno E. Obesity and asthma. J Allergy Clin Immunol. (2018) 141:1169–79. doi: 10.1016/j.jaci.2018.02.004, PMID: 29627041 PMC5973542

[8] Jensen ME Gibson PG Collins CE Wood LG. Airway and systemic inflammation in obese children with asthma. Eur Respir J. (2013) 42:1012–9. doi: 10.1183/09031936.00124912, PMID: 23349447

[9] Huang J Zhou X Dong B Tan H Li Q Zhang J. Obesity-related asthma and its relationship with microbiota. Front Cell Infect Microbiol. (2023) 13:1303899. doi: 10.3389/fcimb.2023.1303899 38292857 PMC10825962

[10] Kim JH Ellwood PE Asher MI. Diet and asthma: looking back, moving forward. Respir Res. (2009) 10:49. doi: 10.1186/1465-9921-10-49, PMID: 19519921 PMC2703624

[11] Nielsen AY Høj S Thomsen SF Meteran H. Vitamin D supplementation for treating atopic dermatitis in children and adults: a systematic review and Meta-analysis. Nutrients. (2024) 16:4128. doi: 10.3390/nu16234128, PMID: 39683522 PMC11644640

[12] Zhang P. The role of diet and nutrition in allergic diseases. Nutrients. (2023) 15:3683. doi: 10.3390/nu15173683, PMID: 37686715 PMC10490368

[13] Hattangdi-Haridas SR Lanham-New SA Wong WHS Ho MHK Darling AL. Vitamin D deficiency and effects of vitamin D supplementation on disease severity in patients with atopic dermatitis: a systematic review and Meta-analysis in adults and children. Nutrients. (2019) 11:1854. doi: 10.3390/nu11081854, PMID: 31405041 PMC6722944

[14] Boggio CMT Veronese F Armari M Zavattaro E Esposto E Savoia P. The Western diet and atopic dermatitis: the potential role of nutrients, contaminants, and additives in dysbiosis and epithelial barrier dysfunction. Antioxidants (Basel). (2025) 14:386. doi: 10.3390/antiox14040386 40298689 PMC12024387

[15] Jiang J Mehrabi Nasab E Athari SM Athari SS. Effects of vitamin E and selenium on allergic rhinitis and asthma pathophysiology. Respir Physiol Neurobiol. (2021) 286:103614. doi: 10.1016/j.resp.2020.103614, PMID: 33422684

[16] Kim SY Sim S Park B Kim JH Choi HG. High-fat and low-carbohydrate diets are associated with allergic rhinitis but not asthma or atopic dermatitis in children. PLoS One. (2016) 11:e0150202. doi: 10.1371/journal.pone.0150202, PMID: 26919190 PMC4769275

[17] Miyake Y Tanaka K Sasaki S Arakawa M. Polyunsaturated fatty acid intake and prevalence of eczema and rhinoconjunctivitis in Japanese children: the Ryukyus child health study. BMC Public Health. (2011) 11:358. doi: 10.1186/1471-2458-11-358, PMID: 21599987 PMC3112140

[18] Molla A. Dietary patterns and their impact on atopic dermatitis: a comprehensive review. Open Dermatol J. (2024) 18. doi: 10.2174/0118743722306189240520075943

[19] Schütte O Bachmann L Shivappa N Hebert JR Felix JF Röder S. Pro-inflammatory diet pictured in children with atopic dermatitis or food allergy: nutritional data of the LiNA cohort. Front Nutr. (2022) 9:2022. doi: 10.3389/fnut.2022.868872 PMC9024336 35464023

[20] Shen H Liao J Zhang L Li P Jiang L Guo T. Association between the dietary inflammatory index and allergic rhinitis results from the national health and nutrition examination survey (2005–2006). J Health Popul Nutr. (2025) 44:179. doi: 10.1186/s41043-025-00932-0, PMID: 40442763 PMC12124075

[21] Ginter E Simko V. Deficiency of vitamin D and vitamin C in the pathogenesis of bronchial asthma. Bratisl Lek Listy. (2016) 117:305–7. doi: 10.4149/bll_2016_060 27546360

[22] Maywald M Rink L. Zinc deficiency and zinc supplementation in allergic diseases. Biomolecules. (2024) 14:863. doi: 10.3390/biom14070863, PMID: 39062576 PMC11274920

[23] Rerksuppaphol S Rerksuppaphol L. Zinc supplementation in children with asthma exacerbation. Pediatr Rep. (2016) 8:6685. doi: 10.4081/pr.2016.6685, PMID: 28058103 PMC5178847

[24] Alwarith J Kahleova H Crosby L Brooks A Brandon L Levin SM. The role of nutrition in asthma prevention and treatment. Nutr Rev. (2020) 78:928–38. doi: 10.1093/nutrit/nuaa005, PMID: 32167552 PMC7550896

[25] Brustad N Bønnelykke K Chawes B. Dietary prevention strategies for childhood asthma. Pediatr Allergy Immunol. (2023) 34:e13984. doi: 10.1111/pai.13984, PMID: 37492917

[26] Cook-Mills JM Avila PC. Vitamin E and D regulation of allergic asthma immunopathogenesis. Int Immunopharmacol. (2014) 23:364–72. doi: 10.1016/j.intimp.2014.08.007, PMID: 25175918 PMC4254328

[27] Koumpagioti D Boutopoulou B Moriki D Priftis KN Douros K. Does adherence to the Mediterranean diet have a protective effect against asthma and allergies in children? A systematic review. Nutrients. (2022) 14:1618. doi: 10.3390/nu14081618, PMID: 35458180 PMC9031000

[28] Tromp IIM Kiefte-de Jong JC de Vries JH Jaddoe VWV Raat H Hofman A. Dietary patterns and respiratory symptoms in pre-school children: the generation R study. Eur Respir J. (2012) 40:681–9. doi: 10.1183/09031936.00119111, PMID: 22362860

[29] Rodríguez-Rodríguez E Perea JM Jiménez AI Rodríguez-Rodríguez P López-Sobaler AM Ortega RM. Fat intake and asthma in Spanish schoolchildren. Eur J Clin Nutr. (2010) 64:1065–71. doi: 10.1038/ejcn.2010.127, PMID: 20664620

[30] Antonio Buendía J Acuña-Cordero R Patiño DG. The role of high carbohydrate-rich food intake and severity of asthma exacerbation in children between 2 to 6 years aged. J Asthma. (2023) 60:412–8. doi: 10.1080/02770903.2022.2062672, PMID: 35389320

[31] Knebusch N Mansour M Vazquez S Coss-Bu JA. Macronutrient and micronutrient intake in children with lung disease. Nutrients. (2023) 15:4142. doi: 10.3390/nu15194142, PMID: 37836425 PMC10574027

[32] Ni D Senior AM Raubenheimer D Simpson SJ Macia L Nanan R. Global associations of macronutrient supply and asthma disease burden. Allergy. (2024) 79:1989–91. doi: 10.1111/all.16067, PMID: 38372164

[33] Antonisamy B Shailesh H Hani Y Ahmed LHM Noor S Ahmed SY. Sphingolipids in childhood asthma and obesity (SOAP study): a protocol of a cross-sectional study. Meta. (2023) 13:1146. doi: 10.3390/metabo13111146, PMID: 37999242 PMC10673587

[34] Shailesh H Noor S Hayati L Belavendra A Van Panhuys N Abou-Samra AB. Asthma and obesity increase inflammatory markers in children. Front Allergy. (2024) 5:1536168. doi: 10.3389/falgy.2024.1536168 39902293 PMC11788363

[35] Wood LG Shivappa N Berthon BS Gibson PG Hebert JR. Dietary inflammatory index is related to asthma risk, lung function and systemic inflammation in asthma. Clin Exp Allergy. (2015) 45:177–83. doi: 10.1111/cea.12323, PMID: 24708388 PMC4190104

[36] Shivappa N Steck SE Hurley TG Hussey JR Hébert JR. Designing and developing a literature-derived, population-based dietary inflammatory index. Public Health Nutr. (2014) 17:1689–96. doi: 10.1017/S1368980013002115, PMID: 23941862 PMC3925198

[37] Public Health England. Government dietary recommendations: Government recommendations for energy and nutrients for males and females aged 1–18 years and 19+ years. London: Public Health England (2016).

[38] Cho SH Jo A Casale T Jeong SJ Hong SJ Cho JK. Soy isoflavones reduce asthma exacerbation in asthmatic patients with high PAI-1-producing genotypes. J Allergy Clin Immunol. (2019) 144:109–17.e4. doi: 10.1016/j.jaci.2019.01.020, PMID: 30707970 PMC6612283

[39] Sharma S Litonjua A. Asthma, allergy, and responses to methyl donor supplements and nutrients. J Allergy Clin Immunol. (2014) 133:1246–54. doi: 10.1016/j.jaci.2013.10.039, PMID: 24360248 PMC4004707

[40] Blatter J Han YY Forno E Brehm J Bodnar L Celedón JC. Folate and asthma. Am J Respir Crit Care Med. (2013) 188:12–7. doi: 10.1164/rccm.201302-0317PP, PMID: 23650899 PMC3735241

[41] Allam MF Lucane RA. Selenium supplementation for asthma. Cochrane Database Syst Rev. (2004) 2004:Cd003538. doi: 10.1002/14651858.CD003538.pub2 15106206 PMC9007145

[42] Xu J Yang L Lin T. Β-Sitosterol targets glucocorticoid receptor to reduce airway inflammation and remodeling in allergic asthma. Pulm Pharmacol Ther. (2023) 78:102183. doi: 10.1016/j.pupt.2022.102183, PMID: 36481301

[43] Mozaffarian D Wu JH. Omega-3 fatty acids and cardiovascular disease: effects on risk factors, molecular pathways, and clinical events. J Am Coll Cardiol. (2011) 58:2047–67. doi: 10.1016/j.jacc.2011.06.063, PMID: 22051327

[44] Calder PC. Omega-3 fatty acids and inflammatory processes: from molecules to man. Biochem Soc Trans. (2017) 45:1105–15. doi: 10.1042/BST20160474, PMID: 28900017

[45] Xie L Atem F Gelfand A Delclos G Messiah SE. Association between asthma and sugar-sweetened beverage consumption in the United States pediatric population. J Asthma. (2022) 59:926–33. doi: 10.1080/02770903.2021.1895210, PMID: 33625285 PMC8846412

[46] Kim HJ Dinh DTT Yang J Herath KHINM Seo SH Son Y-O. High sucrose intake exacerbates airway inflammation through pathogenic Th2 and Th17 response in ovalbumin (OVA)-induced acute allergic asthma in C57BL/6 mice. J Nutr Biochem. (2024) 124:109504. doi: 10.1016/j.jnutbio.2023.109504, PMID: 37944673

[47] Misso NLA Brooks-Wildhaber J Ray S Vally H Thompson PJ. Plasma concentrations of dietary and nondietary antioxidants are low in severe asthma. Eur Respir J. (2005). 26:257–64. 16055873 10.1183/09031936.05.00006705

[48] Berthon BS Macdonald-Wicks LK Gibson PG Wood LG. Investigation of the association between dietary intake, disease severity and airway inflammation in asthma. Respirology. (2013) 18:447–54. doi: 10.1111/resp.12015, PMID: 23145908

[49] Soutar A Seaton A Brown K. Bronchial reactivity and dietary antioxidants. Thorax. (1997) 52:166–70. 9059479 10.1136/thx.52.2.166 PMC1758477

[50] Wood LG Garg ML Gibson PG. A high-fat challenge increases airway inflammation and impairs bronchodilator recovery in asthma. J Allergy Clin Immunol. (2011) 127:1133–40. doi: 10.1016/j.jaci.2011.01.036, PMID: 21377715

[51] Lu C Zhu Y. The dietary inflammatory index and asthma prevalence: a cross-sectional analysis from NHANES. Front Nutr. (2024) 11. doi: 10.3389/fnut.2024.1485399 PMC11622817 39650711

[52] Cilluffo G Han Y-Y Ferrante G Dello Russo M Lauria F Fasola S. The dietary inflammatory index and asthma burden in children: a latent class analysis. Pediatr Allergy Immunol. (2022) 33:e13667. doi: 10.1111/pai.13667, PMID: 34528308 PMC8724457

[53] Han YY Jerschow E Forno E Hua S Mossavar-Rahmani Y Perreira KM. Dietary patterns, asthma, and lung function in the Hispanic community health study/study of Latinos. Ann Am Thorac Soc. (2020) 17:293–301. doi: 10.1513/AnnalsATS.201908-629OC, PMID: 31689128 PMC7044698

[54] Özbey Ü Uçar A Shivappa N Hebert JR. The relationship between dietary inflammatory index, pulmonary functions and asthma control in asthmatics. Iran J Allergy Asthma Immunol. (2019) 18:605–14. doi: 10.18502/ijaai.v18i6.2173, PMID: 32245304

[55] Özbey Yücel Ü Uçar A Sözener Z Balaban S Mungan D Misirligil Z. Effects of dietary intervention on diet inflammatory index and asthma characteristics in obese asthmatic individuals: randomized controlled trial. Eurasian J Pulmonol. (2022) 23:145–151. doi: 10.4103/ejop.ejop_130_20

[56] Pérez-Pérez A Sánchez-Jiménez F Vilariño-García T Sánchez-Margalet V. Role of leptin in inflammation and vice versa. Int J Mol Sci. (2020) 21:5887. doi: 10.3390/ijms21165887, PMID: 32824322 PMC7460646

[57] Malli F Papaioannou AI Gourgoulianis KI Daniil Z. The role of leptin in the respiratory system: an overview. Respir Res. (2010) 11:152. doi: 10.1186/1465-9921-11-152, PMID: 21040518 PMC2988727

[58] Murakami Y Ishii T Nunokawa H Kurata K Narita T Yamashita N. TLR9–IL-2 axis exacerbates allergic asthma by preventing IL-17A hyperproduction. Sci Rep. (2020) 10:18110. doi: 10.1038/s41598-020-75153-y, PMID: 33093516 PMC7581806

[59] Wallace FA Miles EA Evans C Stock TE Yaqoob P Calder PC. Dietary fatty acids influence the production of Th1- but not Th2-type cytokines. J Leukoc Biol. (2001) 69:449–57. doi: 10.1189/jlb.69.3.449, PMID: 11261793

[60] Hall SL Baker T Lajoie S Richgels PK Yang Y McAlees JW. IL-17A enhances IL-13 activity by enhancing IL-13-induced signal transducer and activator of transcription 6 activation. J Allergy Clin Immunol. (2017) 139:462–71. doi: 10.1016/j.jaci.2016.04.037 27417023 PMC5149451

[61] MacLean E Madsen N Vliagoftis H Field C Cameron L. N-3 fatty acids inhibit transcription of human IL-13: implications for development of T helper type 2 immune responses. Br J Nutr. (2013) 109:990–1000. doi: 10.1017/S0007114512002917, PMID: 22849952

[62] Farjadian S Moghtaderi M Kalani M Gholami T Hosseini Teshnizi S. Effects of omega-3 fatty acids on serum levels of T-helper cytokines in children with asthma. Cytokine. (2016) 85:61–6. doi: 10.1016/j.cyto.2016.06.002, PMID: 27288633

[63] Weaver KL Ivester P Seeds M Case LD Arm JP Chilton FH. Effect of dietary fatty acids on inflammatory gene expression in healthy humans. J Biol Chem. (2009) 284:15400–7. doi: 10.1074/jbc.M109.004861, PMID: 19359242 PMC2708836

[64] Gutiérrez S Svahn SL Johansson ME. Effects of Omega-3 fatty acids on immune cells. Int J Mol Sci. (2019) 20:5028. doi: 10.3390/ijms20205028, PMID: 31614433 PMC6834330

[65] Chiurchiù V Leuti A Dalli J Jacobsson A Battistini L Maccarrone M. Proresolving lipid mediators resolvin D1, resolvin D2, and maresin 1 are critical in modulating T cell responses. Sci Transl Med. (2016) 8:353ra111. doi: 10.1126/scitranslmed.aaf7483 PMC5149396 27559094

[66] Ogawa Y Duru EA Ameredes BT. Role of IL-10 in the resolution of airway inflammation. Curr Mol Med. (2008) 8:437–45. doi: 10.2174/156652408785160907, PMID: 18691071 PMC9159958

[67] Chung F. Anti-inflammatory cytokines in asthma and allergy: interleukin-10, interleukin-12, interferon-gamma. Mediat Inflamm. (2001) 10:51–9. doi: 10.1080/09629350120054518, PMID: 11405550 PMC1781697

[68] Borish L Aarons A Rumbyrt J Cvietusa P Negri J Wenzel S. Interleukin-10 regulation in normal subjects and patients with asthma. J Allergy Clin Immunol. (1996) 97:1288–96. 8648025 10.1016/s0091-6749(96)70197-5

[69] Lloyd CM Hawrylowicz CM. Regulatory T cells in asthma. Immunity. (2009) 31:438–49. doi: 10.1016/j.immuni.2009.08.007, PMID: 19766086 PMC3385348

[70] John M Lim S Seybold J Jose P Robichaud A O'Connor B. Inhaled corticosteroids increase interleukin-10 but reduce macrophage inflammatory protein-1alpha, granulocyte-macrophage colony-stimulating factor, and interferon-gamma release from alveolar macrophages in asthma. Am J Respir Crit Care Med. (1998) 157:256–62. 9445307 10.1164/ajrccm.157.1.9703079

[71] Gupta A Dimeloe S Richards DF Chambers ES Black C Urry Z. Defective IL-10 expression and in vitro steroid-induced IL-17A in paediatric severe therapy-resistant asthma. Thorax. (2014) 69:508–15. doi: 10.1136/thoraxjnl-2013-203421, PMID: 24347461

[72] Escalante JH Jones A Sedletsky VV Dooms H. Regulation of IL-10-producing T cells by polyunsaturated fatty acids. J Immunol. (2019) 202:125. doi: 10.4049/jimmunol.202.Supp.125.2

[73] Bradley RL Fisher FF Maratos-Flier E. Dietary fatty acids differentially regulate production of TNF-alpha and IL-10 by murine 3T3-L1 adipocytes. Obesity (Silver Spring). (2008) 16:938–44. doi: 10.1038/oby.2008.39, PMID: 18356844 PMC4862864

[74] Jiménez-Cepeda A Dávila-Said G Orea-Tejeda A González-Islas D Elizondo-Montes M Pérez-Cortes G. Dietary intake of fatty acids and its relationship with FEV1/FVC in patients with chronic obstructive pulmonary disease. Clin Nutrit ESPEN. (2019) 29:92–6. doi: 10.1016/j.clnesp.2018.11.015, PMID: 30661707

[75] McKeever TM Lewis SA Cassano PA Ocké M Burney P Britton J. The relation between dietary intake of individual fatty acids, FEV1 and respiratory disease in Dutch adults. Thorax. (2008) 63:208–14. doi: 10.1136/thx.2007.090399, PMID: 17901161 PMC3979330

[76] Wendell SG Baffi C Holguin F. Fatty acids, inflammation, and asthma. J Allergy Clin Immunol. (2014) 133:1255–64. doi: 10.1016/j.jaci.2013.12.1087, PMID: 24613565 PMC4417548

[77] Okamoto M Mitsunobu F Ashida K Mifune T Hosaki Y Tsugeno H. Effects of dietary supplementation with n-3 fatty acids compared with n-6 fatty acids on bronchial asthma. Intern Med. (2000) 39:107–11. doi: 10.2169/internalmedicine.39.107, PMID: 10732825

[78] Barros R Moreira A Fonseca J Delgado L Graça Castel-Branco M Haahtela T. Dietary intake of α-linolenic acid and low ratio of n-6:n-3 PUFA are associated with decreased exhaled NO and improved asthma control. Br J Nutr. (2011) 106:441–50. doi: 10.1017/S0007114511000328, PMID: 21443816

[79] Brigham EP Woo H McCormack M Rice J Koehler K Vulcain T. Omega-3 and Omega-6 intake modifies asthma severity and response to indoor air pollution in children. Am J Respir Crit Care Med. (2019) 199:1478–86. doi: 10.1164/rccm.201808-1474OC, PMID: 30922077 PMC6580674

[80] Oddy WH de Klerk NH Kendall GE Mihrshahi S Peat JK. Ratio of omega-6 to omega-3 fatty acids and childhood asthma. J Asthma. (2004) 41:319–26. doi: 10.1081/JAS-120026089, PMID: 15260465

[81] Ekström S Sdona E Klevebro S Hallberg J Georgelis A Kull I. Dietary intake and plasma concentrations of PUFAs in childhood and adolescence in relation to asthma and lung function up to adulthood. Am J Clin Nutr. (2022) 115:886–96. doi: 10.1093/ajcn/nqab427, PMID: 34964829 PMC8895221

[82] Kan H Stevens J Heiss G Rose KM London SJ. Dietary fiber, lung function, and chronic obstructive pulmonary disease in the atherosclerosis risk in communities study. Am J Epidemiol. (2008) 167:570–8. doi: 10.1093/aje/kwm343 18063592 PMC2377022

[83] Hanson C Lyden E Rennard S Mannino DM Rutten EP Hopkins R. The relationship between dietary fiber intake and lung function in the National Health and nutrition examination surveys. Ann Am Thorac Soc. (2016) 13:643–50. doi: 10.1513/AnnalsATS.201509-609OC, PMID: 26783997

[84] Reynolds A Mann J Cummings J Winter N Mete E Te Morenga L. Carbohydrate quality and human health: a series of systematic reviews and meta-analyses. Lancet. (2019) 393:434–45. doi: 10.1016/S0140-6736(18)31809-9, PMID: 30638909

[85] Soliman GA. Dietary Fiber, atherosclerosis, and cardiovascular disease. Nutrients. (2019) 11:1155. doi: 10.3390/nu11051155, PMID: 31126110 PMC6566984

[86] Ma Y Griffith JA Chasan-Taber L Olendzki BC Jackson E Stanek EJ 3rd. Association between dietary fiber and serum C-reactive protein. Am J Clin Nutr. (2006) 83:760–6. doi: 10.1093/ajcn/83.4.760, PMID: 16600925 PMC1456807

[87] Ma Y Hébert JR Li W Bertone-Johnson ER Olendzki B Pagoto SL. Association between dietary fiber and markers of systemic inflammation in the Women's Health Initiative observational study. Nutrition. (2008) 24:941–9. doi: 10.1016/j.nut.2008.04.005, PMID: 18562168 PMC2603616

[88] Fu J Zheng Y Gao Y Xu W. Dietary Fiber intake and gut microbiota in human health. Microorganisms. (2022) 10:2507. doi: 10.3390/microorganisms10122507, PMID: 36557760 PMC9787832

[89] Trompette A Gollwitzer ES Yadava K Sichelstiel AK Sprenger N Ngom-Bru C. Gut microbiota metabolism of dietary fiber influences allergic airway disease and hematopoiesis. Nat Med. (2014) 20:159–66. doi: 10.1038/nm.3444, PMID: 24390308

[90] Wang Z Lai Z Zhang X Huang P Xie J Jiang Q. Altered gut microbiome compositions are associated with the severity of asthma. J Thorac Dis. (2021) 13:4322–38. doi: 10.21037/jtd-20-2189, PMID: 34422359 PMC8339736

[91] Hilty M Burke C Pedro H Cardenas P Bush A Bossley C. Disordered microbial communities in asthmatic airways. PLoS One. (2010) 5:e8578. doi: 10.1371/journal.pone.0008578, PMID: 20052417 PMC2798952

[92] Loverdos K Bellos G Kokolatou L Vasileiadis I Giamarellos E Pecchiari M. Lung microbiome in asthma: current perspectives. J Clin Med. (2019) 8:1967. doi: 10.3390/jcm8111967, PMID: 31739446 PMC6912699

[93] Durack J Lynch SV Nariya S Bhakta NR Beigelman A Castro M. Features of the bronchial bacterial microbiome associated with atopy, asthma, and responsiveness to inhaled corticosteroid treatment. J Allergy Clin Immunol. (2017) 140:63–75. doi: 10.1016/j.jaci.2016.08.055, PMID: 27838347 PMC5502827

[94] Anand S Mande SS. Diet, microbiota and gut-lung connection. Front Microbiol. (2018) 9:2147. doi: 10.3389/fmicb.2018.02147, PMID: 30283410 PMC6156521

[95] Lu KD Forno E. Exercise and lifestyle changes in pediatric asthma. Curr Opin Pulm Med. (2020) 26:103–11. doi: 10.1097/MCP.0000000000000636, PMID: 31652153 PMC7094764

