# Supplementary material for: Dietary fiber and fatty acids may impact clinical outcomes in pediatric obesity-associated asthma: insights from the SOAP study
Source: Front Nutr. 2025 Nov 3;12:1687082. doi: 10.3389/fnut.2025.1687082 (PMC12620195; doi:10.3389/fnut.2025.1687082)
Supplement: Supplementary file 1 [file Data_Sheet_1.PDF]

# *Supplementary Material*

## 1 Supplementary Tables

### Supplementary Table 1: Analysis of nutrient intake and diet quality across the four study groups

| Nutrient | NW-A | OO-A | NW | OO | p-value | Pairwise comparison (p-value) |
|---|---|---|---|---|---|---|
| **Energy (kcal)[a]** | 1587 (1293-1928) | 1519 (1308-1971) | 1517 (1195-1786) | 1361 (995-1745) | ns[#] | ns |
| **Protein (g)[a]** | 55.8 (49.5-71.0) | 63.6 (52.4-79.2) | 58.6 (48.3-77.2) | 54.6 (47.1-80.0) | ns[#] | ns |
| **Carbohydrate (g)[b]** | 192 (48.8) | 193 (63.3) | 178 (51.3) | 176 (79.4) | ns* | ns |
| **Total fat (g)[a]** | 65.1 (49.7-77.1) | 62.7 (48.5-74.4) | 57.2 (45.3-78.1) | 56.0 (41.2-63.2) | ns[#] | ns |
| **Saturated fat (g)[b]** | 19.7 (7.00) | 18.5 (7.24) | 19.7 (8.43) | 18.6 (7.02) | ns* | ns |
| **Total MUFA (g)[a]** | 18.0 (10.6-21.4) | 17.6 (11.1-22.1) | 15.5 (12.0-25.3) | 15.0 (12.4-18.8) | ns[#] | ns |
| **Total PUFA (g)[a]** | 9.10 (6.58-12.3) | 9.41 (5.67-16.2) | 9.04 (7.56-14.2) | 8.17 (5.88-11.0) | ns[#] | ns |
| **Total fiber (g)[a]** | 8.26 (7.17-13.7) | 9.75 (8.18-11.8) | 11.1 (8.28-13.5) | 11.4 (6.99-17.8) | ns[#] | ns |
| **Added sugar (g)[a]** | 14.1 (2.3-23.4) | 6.07 (0.11-13.27) | 5.2 (0.00-9.27) | 0.00 (0.0-6.0) | 0.008[#] | NW-A vs. OO (p=0.006) |
| **PUFA 18:4 (g)[a]** | 0.0003 (0.00-0.0008) | 0.000 (0.00-0.002) | 0.0017 (0.0008-0.0033) | 0.0003 (0.00-0.0012) | <0.001[#] | NW-A vs. NW (p=0.007) OO-A vs. NW (p=0.01) NW vs. OO (p=0.015) |
| **PUFA 22:5 (g)[a]** | 0.004 (0.0014-0.01) | 0.0088(0.0058- 0.016) | 0.01 (0.005-0.018) | 0.009 (0.003- 0.02) | 0.049[#] | NW-A vs. NW (p=0.033) |
| **PUFA 20:4 (g)[a]** | 0.037 (0.024-0.081) | 0.086 (0.058- 0.14) | 0.085 (0.057-0.128) | 0.079 (0.048- 0.14) | 0.034[#] | NW-A vs. NW (p=0.041) |
| **n-6/n-3 PUFA ratio[a]** | 8.96 (8.16-10.22) | 8.23 (7.56-9.59) | 7.72 (7.23-9.48) | 8.44 (6.78-9.86) | ns[#] | ns |
| **Fluoride (µg)[a]** | 18.6 (7.7-58.5) | 85.6 (14.7-245.8) | 113 (36.4-245.3) | 67.8 (23.3-388.7) | 0.036[#] | NW-A vs. NW (p=0.025) |
| **Selenium (µg)[a]** | 41.4 (33.1-52.1) | 72.2 (54.3-88.9) | 70.3 (51.2-94.6) | 60.6 (45.9-83.9) | 0.003[#] | NW-A vs. OO-A (p=0.008) NW-A vs. NW (p=0.003) |
| **Vitamin E (mg)[a]** | 1.09 (0.7-2.0) | 0.49 (0.069-1.2) | 0.23 (0.01-0.82)) | 0.18 (0.01-0.86) | 0.004[#] | NW-A vs. NW (p=0.004) |

| | | | | | | |
|---|---|---|---|---|---|---|
| | | | | | | NW-A vs. OO (p=0.009) |
| **Folic acid (µg)[a]** | 43.3(27.4-60.9) | 97.3 (I46.3-131.6 | 81.8 (42.4-136.8) | 74.7 (22.6-114.6) | 0.006[#] | NW-A vs. OO-A (p=0.019) NW-A vs. NW (p=0.03) |
| **Folate, total (µg)[a]** | 162.5 (109.9-217.2) | 266.8 (164.2-319.8) | 217.8 (179.1-328.3) | 254.2 (130-327.1) | 0.027[#] | NW-A vs. NW (p=0.027) |
| **Folate, DFE (µg)[b]** | 206 (88.6) | 313 (131.1) | 312 (131.9) | 280 (136.9) | 0.034* | NW-A vs. OO-A (p=0.045) NW-A vs. NW (p=0.035) |
| **Glutamic acid (mg)[b]** | 3586 (2013) | 6179 (3346) | 5415 (2735) | 5092 (2377) | 0.032* | NW-A vs. OO-A (p=0.018) |
| **β-Sitosterol (mg)[a]** | 0.12 (0.018-1.71) | 0.06733 (0.00-5.93) | 0.053 (0.000-0.279) | 0.00 (0.00-0.04) | 0.019[#] | NW-A vs. OO (p=0.039) |
| **Isoflavones (mg)[a]** | 0.00 (0.00-0.004) | 0.007 (0.0013-0.048) | 0.016 (0.004-0.083) | 0.009 (0.002-0.044) | 0.002[#] | NW-A vs. NW (p=0.001) NW-A vs. OO (p=0.022) |
| **Betaine (mg)[a]** | 1.07 (0.25-5.14) | 7.96 (2.12-35.9) | 9.63 (2.81-37.6) | 12.25 (2.01-24.8) | 0.01[#] | NW-A vs. OO-A (p=0.027) NW-A vs. NW (p=0.008) |
| **DII score[a]** | 3.24 (2.68-3.84) | 2.86 (2.37-3.57) | 2.69 (1.91-3.41) | 2.94 (1.88-3.46) | ns[#] | ns |

*Note: [a]Median (IQR), [b]Mean (SD). Group comparisons performed via ANOVA (*) or the Kruskal-Wallis test ([#]) based on data distribution, followed by the Tukey's honestly significant difference or Bonferroni-corrected Dunn's post hoc test respectively. Only nutrients with significant overall group differences are shown (p<0.05). Abbreviations: DFE, dietary folate equivalents; DII, dietary inflammatory index; MUFA, monounsaturated fatty acids; ns, not significant; NW, normal weight; NW-A, normal weight with asthma; OO, overweight or obesity; OO-A, overweight or obesity with asthma; PUFA, polyunsaturated fatty acid/s.*

## Supplementary Table 2: Statistical summary of the ridge regression analysis to identify the top-ranking nutrients associated with asthma and obesity-associated asthma

| Nutrient | Coefficient | AUC | Combined score | Combined score (signed) |
|---|---|---|---|---|
| | | **NW-A vs. OO-A** | | |
| **PUFA 22:5 (g)** | -3.42959 | 0.70072 | 2.40319 | -2.40319 |
| **MUFA 22:1 (g)** | -2.59940 | 0.64183 | 1.66837 | -1.66837 |
| **PUFA 20:5 (g)** | -2.04644 | 0.71875 | 1.47088 | -1.47088 |
| **Total isoflavones (mg)** | -1.09519 | 0.74038 | 0.81086 | -0.81086 |
| **MUFA 14:1 (g)** | -0.98644 | 0.66947 | 0.66039 | -0.66039 |
| **SFA 15:0 (g)** | -0.90646 | 0.69471 | 0.62973 | -0.62973 |
| **SFA 24:0 (g)** | -0.82629 | 0.58894 | 0.48664 | -0.48664 |
| **PUFA 22:6 (g)** | -0.54207 | 0.70313 | 0.38114 | -0.38114 |
| **SFA 17:0 (g)** | -0.53634 | 0.64303 | 0.34488 | -0.34488 |

| | | | | |
|---|---|---|---|---|
| **SFA 22:0 (g)** | -0.49064 | 0.62500 | 0.30665 | -0.30665 |
| **γ-Tocotrienol (mg)** | -0.50884 | 0.56611 | 0.28806 | -0.28806 |
| **PUFA 20:4 (g)** | -0.37447 | 0.73197 | 0.27410 | -0.27410 |
| **Chromium (mg)** | 0.31273 | 0.60216 | 0.18832 | 0.18832 |
| **SFA 20:0 (g)** | -0.31831 | 0.59135 | 0.18823 | -0.18823 |
| **α-Tocotrienol (mg)** | -0.22827 | 0.65625 | 0.14980 | -0.14980 |
| **MUFA 20:1 (g)** | -0.17121 | 0.62500 | 0.10700 | -0.10700 |
| **MUFA 16:1 (g)** | -0.09275 | 0.71635 | 0.06644 | -0.06644 |
| **Galactose (g)** | 0.09385 | 0.63341 | 0.05945 | 0.05945 |
| **Thiamin (mg)** | -0.05236 | 0.67548 | 0.03537 | -0.03537 |
| **β-Tocotrienol (mg)** | -0.05791 | 0.54447 | 0.03153 | -0.03153 |
| **Pyridoxine (vitamin B6) (mg)** | 0.05933 | 0.51442 | 0.03052 | 0.03052 |
| **β-Tocopherol (mg)** | -0.04751 | 0.62260 | 0.02958 | -0.02958 |
| **SFA 12:0 (g)** | 0.04170 | 0.58894 | 0.02456 | 0.02456 |
| **Vitamin E (mg)** | 0.02854 | 0.72837 | 0.02078 | 0.02078 |
| **PUFA 18:3 (g)** | -0.03007 | 0.66106 | 0.01988 | -0.01988 |
| **Vitamin E (IU)** | 0.01916 | 0.72837 | 0.01396 | 0.01396 |
| **Insoluble fiber (g)** | 0.01923 | 0.54567 | 0.01049 | 0.01049 |
| **SFA 8:0 (g)** | 0.01935 | 0.52043 | 0.01007 | 0.01007 |
| **Soluble fiber (g)** | 0.01394 | 0.55168 | 0.00769 | 0.00769 |
| **SFA 4:0 (g)** | 0.01322 | 0.53245 | 0.00704 | 0.00704 |
| **SFA 18:0 (g)** | -0.00960 | 0.67308 | 0.00646 | -0.00646 |
| **Trans fatty acids (g)** | -0.01190 | 0.52163 | 0.00621 | -0.00621 |
| **Crude fiber (g)** | 0.00907 | 0.62740 | 0.00569 | 0.00569 |
| **Copper (mg)** | 0.00952 | 0.52404 | 0.00499 | 0.00499 |
| **Zinc (mg)** | -0.00743 | 0.64183 | 0.00477 | -0.00477 |
| **δ-Tocopherol (mg)** | 0.00784 | 0.57332 | 0.00449 | 0.00449 |
| **SFA 14:0 (g)** | -0.00671 | 0.57452 | 0.00386 | -0.00386 |
| **Vitamin D (µg)** | -0.00663 | 0.56971 | 0.00378 | -0.00378 |
| **SFA 10:0 (g)** | 0.00678 | 0.50962 | 0.00345 | 0.00345 |
| **Molybdenum (µg)** | -0.00496 | 0.67188 | 0.00333 | -0.00333 |
| **Vitamin E (α-tocopherol) (mg)** | 0.00623 | 0.51442 | 0.00321 | 0.00321 |
| **Total fiber (g)** | 0.00474 | 0.54808 | 0.00260 | 0.00260 |
| **Cobalamin (vitamin B12) (µg)** | -0.00435 | 0.57933 | 0.00252 | -0.00252 |
| **Salt (g)** | -0.00347 | 0.60096 | 0.00209 | -0.00209 |
| **SFA 16:0 (g)** | -0.00294 | 0.67788 | 0.00200 | -0.00200 |
| **Total PUFA (g)** | 0.00370 | 0.53606 | 0.00198 | 0.00198 |
| **Total SFA (g)** | 0.00327 | 0.56731 | 0.00186 | 0.00186 |
| **Added sugar (g)** | 0.00266 | 0.63702 | 0.00169 | 0.00169 |
| **Betaine (mg)** | -0.00219 | 0.75240 | 0.00164 | -0.00164 |
| **Biotin (µg)** | 0.00301 | 0.51442 | 0.00155 | 0.00155 |

| | | | | |
|---|---|---|---|---|
| **SFA 6:0 (g)** | -0.00277 | 0.53846 | 0.00149 | -0.00149 |
| **Fructose (g)** | 0.00257 | 0.56250 | 0.00145 | 0.00145 |
| **Glucose (g)** | 0.00263 | 0.54327 | 0.00143 | 0.00143 |
| **Riboflavin (mg)** | -0.00185 | 0.60337 | 0.00112 | -0.00112 |
| **β-Sitosterol (mg)** | 0.00214 | 0.52163 | 0.00112 | 0.00112 |
| **PUFA 18:2 (g)** | 0.00192 | 0.57692 | 0.00111 | 0.00111 |
| **Sucrose (g)** | 0.00199 | 0.53125 | 0.00106 | 0.00106 |
| **Selenium (μg)** | -0.00137 | 0.75721 | 0.00103 | -0.00103 |
| **Lactose (g)** | -0.00170 | 0.55649 | 0.00095 | -0.00095 |
| **γ-Tocopherol (mg)** | 0.00154 | 0.58894 | 0.00091 | 0.00091 |
| **Manganese (mg)** | -0.00155 | 0.57692 | 0.00089 | -0.00089 |
| **Iodine (μg)** | 0.00128 | 0.66466 | 0.00085 | 0.00085 |
| **Total fat (g)** | 0.00143 | 0.54087 | 0.00077 | 0.00077 |
| **Folic acid (μg)** | -0.00098 | 0.78125 | 0.00077 | -0.00077 |
| **Niacin (mg)** | -0.00113 | 0.63942 | 0.00072 | -0.00072 |
| **Total sugar (g)** | 0.00113 | 0.61779 | 0.00070 | 0.00070 |
| **Theobromine (mg)** | 0.00102 | 0.57813 | 0.00059 | 0.00059 |
| **Niacin equivalents (mg)** | -0.00063 | 0.66106 | 0.00041 | -0.00041 |
| **MUFA 18:1 (g)** | -0.00066 | 0.60577 | 0.00040 | -0.00040 |
| **Food folate (μg)** | -0.00051 | 0.69712 | 0.00035 | -0.00035 |
| **Protein (g)** | -0.00038 | 0.60337 | 0.00023 | -0.00023 |
| **Vitamin D (IU)** | 0.00035 | 0.62981 | 0.00022 | 0.00022 |
| **Phytosterols (mg)** | 0.00036 | 0.57933 | 0.00021 | 0.00021 |
| **Total folate (μg)** | -0.00025 | 0.74038 | 0.00019 | -0.00019 |
| **Folate, DFE (μg)** | -0.00024 | 0.75000 | 0.00018 | -0.00018 |
| **Choline (mg)** | -0.00024 | 0.68990 | 0.00017 | -0.00017 |
| **Iron (mg)** | -0.00023 | 0.60577 | 0.00014 | -0.00014 |
| **Fluoride (μg)** | -0.00019 | 0.68029 | 0.00013 | -0.00013 |
| **Total trans and saturated fat (g)** | -0.00021 | 0.53365 | 0.00011 | -0.00011 |
| **Vitamin C (mg)** | 0.00020 | 0.54087 | 0.00011 | 0.00011 |
| **Caffeine (mg)** | -0.00018 | 0.52764 | 0.00010 | -0.00010 |
| **Calcium (mg)** | 0.00012 | 0.59615 | 0.00007 | 0.00007 |
| **Tryptophan (mg)** | -0.00008 | 0.73317 | 0.00006 | -0.00006 |
| **β-Cryptoxanthin (μg)** | -0.00011 | 0.56731 | 0.00006 | -0.00006 |
| **Magnesium (mg)** | 0.00010 | 0.59135 | 0.00006 | 0.00006 |
| **Chloride (mg)** | 0.00009 | 0.60096 | 0.00005 | 0.00005 |
| **Carbohydrate (g)** | 0.00009 | 0.52404 | 0.00005 | 0.00005 |
| **NW-A vs. NW** | | | | |
| **PUFA 18:4 (g)** | -13.26926 | 0.83832 | 11.12383 | -11.12383 |
| **PUFA 22:5 (g)** | -5.80351 | 0.72554 | 4.21070 | -4.21070 |
| **MUFA 22:1 (g)** | -3.17592 | 0.65625 | 2.08420 | -2.08420 |

| | | | | |
|---|---|---|---|---|
| **Total isoflavones (mg)** | -1.20466 | 0.88043 | 1.06062 | -1.06062 |
| **PUFA 20:5 (g)** | -1.15642 | 0.69973 | 0.80918 | -0.80918 |
| **MUFA 14:1 (g)** | -0.98353 | 0.61957 | 0.60936 | -0.60936 |
| **SFA 15:0 (g)** | -0.87728 | 0.67391 | 0.59121 | -0.59121 |
| **SFA 24:0 (g)** | -0.92684 | 0.50951 | 0.47224 | -0.47224 |
| **PUFA 22:6 (g)** | -0.61556 | 0.66984 | 0.41233 | -0.41233 |
| **γ-Tocotrienol (mg)** | -0.64962 | 0.56114 | 0.36453 | -0.36453 |
| **PUFA 20:4 (g)** | -0.48430 | 0.73913 | 0.35796 | -0.35796 |
| **MUFA 20:1 (g)** | -0.48423 | 0.70652 | 0.34212 | -0.34212 |
| **β-Tocopherol (mg)** | 0.49758 | 0.58016 | 0.28868 | 0.28868 |
| **SFA 20:0 (g)** | 0.25936 | 0.55978 | 0.14519 | 0.14519 |
| **Thiamin (mg)** | -0.13288 | 0.71739 | 0.09533 | -0.09533 |
| **α-Tocotrienol (mg)** | -0.13981 | 0.60462 | 0.08453 | -0.08453 |
| **SFA 6:0 (g)** | -0.12257 | 0.65217 | 0.07994 | -0.07994 |
| **MUFA 16:1 (g)** | -0.09251 | 0.68207 | 0.06310 | -0.06310 |
| **Chromium (mg)** | -0.10335 | 0.59375 | 0.06136 | -0.06136 |
| **PUFA 18:3 (g)** | -0.07823 | 0.69565 | 0.05442 | -0.05442 |
| **Pyridoxine (vitamin B6) (mg)** | 0.08499 | 0.61141 | 0.05196 | 0.05196 |
| **SFA 8:0 (g)** | -0.08659 | 0.58424 | 0.05059 | -0.05059 |
| **Galactose (g)** | 0.06366 | 0.55978 | 0.03563 | 0.03563 |
| **SFA 4:0 (g)** | -0.05349 | 0.63451 | 0.03394 | -0.03394 |
| **SFA 14:0 (g)** | -0.04639 | 0.67120 | 0.03114 | -0.03114 |
| **SFA 10:0 (g)** | -0.04933 | 0.62500 | 0.03083 | -0.03083 |
| **SFA 12:0 (g)** | 0.03854 | 0.55163 | 0.02126 | 0.02126 |
| **Vitamin E (mg)** | 0.02325 | 0.81522 | 0.01895 | 0.01895 |
| **Soluble fiber (g)** | -0.03194 | 0.52717 | 0.01684 | -0.01684 |
| **Maltose (g)** | 0.02553 | 0.52446 | 0.01339 | 0.01339 |
| **Vitamin E (IU)** | 0.01560 | 0.81522 | 0.01272 | 0.01272 |
| **SFA 18:0 (g)** | -0.01486 | 0.60870 | 0.00905 | -0.00905 |
| **β-Sitosterol (mg)** | 0.01191 | 0.73370 | 0.00874 | 0.00874 |
| **Riboflavin (mg)** | -0.01354 | 0.53804 | 0.00728 | -0.00728 |
| **Vitamin E (α-tocopherol) (mg)** | 0.01382 | 0.51087 | 0.00706 | 0.00706 |
| **Pantothenic acid (mg)** | 0.01262 | 0.52989 | 0.00669 | 0.00669 |
| **Vitamin D (μg)** | -0.01060 | 0.58696 | 0.00622 | -0.00622 |
| **SFA 16:0 (g)** | -0.00867 | 0.66304 | 0.00575 | -0.00575 |
| **SFA 22:0 (g)** | 0.01017 | 0.51902 | 0.00528 | 0.00528 |
| **Total trans and saturated fat (g)** | 0.00754 | 0.59783 | 0.00451 | 0.00451 |
| **Crude fiber (g)** | -0.00690 | 0.58152 | 0.00401 | -0.00401 |
| **Zinc (mg)** | -0.00602 | 0.58424 | 0.00351 | -0.00351 |
| **δ-Tocopherol (mg)** | 0.00579 | 0.60054 | 0.00348 | 0.00348 |
| **Iodine (μg)** | 0.00387 | 0.72011 | 0.00278 | 0.00278 |

| | | | | |
|---|---|---|---|---|
| **Insoluble fiber (g)** | 0.00445 | 0.53261 | 0.00237 | 0.00237 |
| **γ-Tocopherol (mg)** | 0.00396 | 0.59783 | 0.00237 | 0.00237 |
| **Added sugar (g)** | 0.00294 | 0.72283 | 0.00213 | 0.00213 |
| **Selenium (µg)** | -0.00231 | 0.83424 | 0.00192 | -0.00192 |
| **Betaine (mg)** | -0.00244 | 0.77989 | 0.00190 | -0.00190 |
| **Biotin (µg)** | 0.00290 | 0.55978 | 0.00162 | 0.00162 |
| **Total SFA (g)** | 0.00275 | 0.56250 | 0.00155 | 0.00155 |
| **Niacin (mg)** | -0.00241 | 0.58967 | 0.00142 | -0.00142 |
| **Sucrose (g)** | 0.00239 | 0.52446 | 0.00125 | 0.00125 |
| **Total fat (g)** | 0.00186 | 0.61685 | 0.00115 | 0.00115 |
| **Total MUFA (g)** | 0.00193 | 0.54891 | 0.00106 | 0.00106 |
| **Salt (g)** | -0.00177 | 0.55435 | 0.00098 | -0.00098 |
| **Folic acid (µg)** | -0.00103 | 0.80435 | 0.00083 | -0.00083 |
| **MUFA 18:1 (g)** | 0.00139 | 0.56250 | 0.00078 | 0.00078 |
| **Lactose (g)** | -0.00123 | 0.55707 | 0.00068 | -0.00068 |
| **Niacin equivalents (mg)** | -0.00114 | 0.58696 | 0.00067 | -0.00067 |
| **Theobromine (mg)** | 0.00086 | 0.61277 | 0.00052 | 0.00052 |
| **Fructose (g)** | -0.00082 | 0.60598 | 0.00050 | -0.00050 |
| **Total sugar (g)** | 0.00083 | 0.58967 | 0.00049 | 0.00049 |
| **Glucose (g)** | 0.00067 | 0.53533 | 0.00036 | 0.00036 |
| **Caffeine (mg)** | -0.00056 | 0.63179 | 0.00035 | -0.00035 |
| **Molybdenum (µg)** | -0.00064 | 0.52853 | 0.00034 | -0.00034 |
| **Carbohydrate (g)** | 0.00054 | 0.58967 | 0.00032 | 0.00032 |
| **PUFA 18:2 (g)** | -0.00053 | 0.56522 | 0.00030 | -0.00030 |
| **Available carbohydrate (g)** | -0.00044 | 0.66033 | 0.00029 | -0.00029 |
| **Food folate (µg)** | -0.00042 | 0.63859 | 0.00027 | -0.00027 |
| **Total folate (µg)** | -0.00032 | 0.75000 | 0.00024 | -0.00024 |
| **Vitamin D (IU)** | 0.00036 | 0.61413 | 0.00022 | 0.00022 |
| **Folate, DFE (µg)** | -0.00026 | 0.73098 | 0.00019 | -0.00019 |
| **Choline (mg)** | -0.00020 | 0.64402 | 0.00013 | -0.00013 |
| **Phosphorus (mg)** | -0.00014 | 0.67935 | 0.00010 | -0.00010 |
| **Total fiber (g)** | -0.00018 | 0.54620 | 0.00010 | -0.00010 |
| **Fluoride (µg)** | -0.00011 | 0.70924 | 0.00008 | -0.00008 |
| **Tryptophan (mg)** | -0.00011 | 0.67120 | 0.00007 | -0.00007 |
| **Energy (kcal)** | 0.00010 | 0.62772 | 0.00006 | 0.00006 |
| **Phytosterols (mg)** | 0.00011 | 0.54620 | 0.00006 | 0.00006 |
| **Vitamin K (µg)** | 0.00009 | 0.52174 | 0.00005 | 0.00005 |
| **OO-A vs. OO** | | | | |
| **PUFA 22:5 (g)** | -0.046554 | 0.574519 | 0.026746 | -0.026746 |
| **PUFA 18:4 (g)** | -0.044254 | 0.591346 | 0.026169 | -0.026169 |
| **Chromium (mg)** | -0.019496 | 0.640625 | 0.012489 | -0.012489 |

| | | | | |
|---|---|---|---|---|
| **γ-Tocotrienol (mg)** | 0.011488 | 0.536058 | 0.006158 | 0.006158 |
| **PUFA 20:5 (g)** | -0.009277 | 0.510817 | 0.004739 | -0.004739 |
| **α-Tocotrienol (mg)** | -0.008099 | 0.561298 | 0.004546 | -0.004546 |
| **MUFA 14:1 (g)** | -0.007223 | 0.542067 | 0.003916 | -0.003916 |
| **SFA 15:0 (g)** | -0.007152 | 0.530048 | 0.003791 | -0.003791 |
| **Total isoflavones (mg)** | -0.006134 | 0.570913 | 0.003502 | -0.003502 |
| **β-Tocopherol (mg)** | 0.005811 | 0.580529 | 0.003374 | 0.003374 |
| **SFA 20:0 (g)** | 0.004627 | 0.587740 | 0.002720 | 0.002720 |
| **SFA 22:0 (g)** | 0.004480 | 0.586538 | 0.002628 | 0.002628 |
| **β-Tocotrienol (mg)** | 0.004397 | 0.512019 | 0.002251 | 0.002251 |
| **Galactose (g)** | -0.002981 | 0.575721 | 0.001716 | -0.001716 |
| **Thiamin (mg)** | 0.001481 | 0.626202 | 0.000927 | 0.000927 |
| **δ-Tocopherol (mg)** | 0.001327 | 0.681490 | 0.000904 | 0.000904 |
| **SFA 6:0 (g)** | -0.000983 | 0.603365 | 0.000593 | -0.000593 |
| **SFA 4:0 (g)** | -0.000814 | 0.610577 | 0.000497 | -0.000497 |
| **Crude fiber (g)** | -0.000762 | 0.646635 | 0.000493 | -0.000493 |
| **MUFA 16:1 (g)** | -0.000736 | 0.550481 | 0.000405 | -0.000405 |
| **SFA 10:0 (g)** | -0.000567 | 0.603365 | 0.000342 | -0.000342 |
| **PUFA 18:3 (g)** | 0.000463 | 0.598558 | 0.000277 | 0.000277 |
| **SFA 17:0 (g)** | -0.000477 | 0.503606 | 0.000240 | -0.000240 |
| **Manganese (mg)** | 0.000340 | 0.550481 | 0.000187 | 0.000187 |
| **Pyridoxine (vitamin B6) (mg)** | -0.000344 | 0.543269 | 0.000187 | -0.000187 |
| **SFA 24:0 (g)** | -0.000322 | 0.551683 | 0.000178 | -0.000178 |
| **Pantothenic acid (mg)** | -0.000271 | 0.584135 | 0.000158 | -0.000158 |
| **SFA 14:0 (g)** | -0.000244 | 0.586538 | 0.000143 | -0.000143 |
| **Trans fatty acids (g)** | 0.000216 | 0.558894 | 0.000121 | 0.000121 |
| **γ-Tocopherol (mg)** | 0.000192 | 0.627404 | 0.000121 | 0.000121 |
| **PUFA 20:4 (g)** | -0.000229 | 0.525240 | 0.000120 | -0.000120 |
| **β-Sitosterol (mg)** | 0.000125 | 0.735577 | 0.000092 | 0.000092 |
| **Fructose (g)** | -0.000130 | 0.677885 | 0.000088 | -0.000088 |
| **Total fiber (g)** | -0.000138 | 0.632212 | 0.000087 | -0.000087 |
| **Glucose (g)** | -0.000103 | 0.598558 | 0.000062 | -0.000062 |
| **Cobalamin (vitamin B12) (µg)** | 0.000092 | 0.576923 | 0.000053 | 0.000053 |
| **Added sugar (g)** | 0.000066 | 0.647837 | 0.000043 | 0.000043 |
| **PUFA 18:2 (g)** | 0.000075 | 0.564904 | 0.000042 | 0.000042 |
| **Total trans and saturated fat (g)** | 0.000066 | 0.620192 | 0.000041 | 0.000041 |
| **Biotin (µg)** | -0.000060 | 0.600962 | 0.000036 | -0.000036 |
| **Zinc (mg)** | -0.000064 | 0.557692 | 0.000035 | -0.000035 |
| **Vitamin E (α-tocopherol) (mg)** | -0.000065 | 0.538462 | 0.000035 | -0.000035 |
| **Iron (mg)** | 0.000060 | 0.579327 | 0.000035 | 0.000035 |
| **Salt (g)** | -0.000063 | 0.519231 | 0.000032 | -0.000032 |

| | | | | |
|---|---|---|---|---|
| **Total PUFA (g)** | 0.000054 | 0.552885 | 0.000030 | 0.000030 |
| **Phytosterols (mg)** | -0.000035 | 0.680288 | 0.000024 | -0.000024 |
| **Vitamin D (µg)** | 0.000043 | 0.514423 | 0.000022 | 0.000022 |
| **Niacin equivalents (mg)** | 0.000037 | 0.536058 | 0.000020 | 0.000020 |
| **Iodine (µg)** | 0.000028 | 0.550481 | 0.000015 | 0.000015 |
| **SFA 18:0 (g)** | 0.000017 | 0.536058 | 0.000009 | 0.000009 |
| **Lactose (g)** | 0.000015 | 0.525240 | 0.000008 | 0.000008 |
| **Total fat (g)** | 0.000012 | 0.593750 | 0.000007 | 0.000007 |
| **Folic acid (µg)** | 0.000010 | 0.680288 | 0.000007 | 0.000007 |
| **Vitamin E (mg)** | -0.000009 | 0.631010 | 0.000006 | -0.000006 |
| **Molybdenum (µg)** | 0.000009 | 0.610577 | 0.000006 | 0.000006 |
| **Available carbohydrate (g)** | -0.000009 | 0.568510 | 0.000005 | -0.000005 |
| **Selenium (µg)** | 0.000008 | 0.548077 | 0.000005 | 0.000005 |
| **Theobromine (mg)** | -0.000008 | 0.528846 | 0.000004 | -0.000004 |
| **Vitamin E (IU)** | -0.000006 | 0.633413 | 0.000004 | -0.000004 |
| **Carbohydrate (g)** | 0.000005 | 0.625000 | 0.000003 | 0.000003 |
| **Caffeine (mg)** | 0.000006 | 0.555288 | 0.000003 | 0.000003 |
| **Niacin (mg)** | 0.000005 | 0.512019 | 0.000003 | 0.000003 |
| **Total sugar (g)** | -0.000003 | 0.557692 | 0.000002 | -0.000002 |
| **Vitamin D (IU)** | 0.000003 | 0.562500 | 0.000002 | 0.000002 |
| **Choline (mg)** | -0.000003 | 0.560096 | 0.000002 | -0.000002 |
| **Folate, DFE (µg)** | 0.000003 | 0.637019 | 0.000002 | 0.000002 |
| **Cholesterol (mg)** | -0.000003 | 0.552885 | 0.000002 | -0.000002 |
| **Total SFA (g)** | -0.000003 | 0.514423 | 0.000001 | -0.000001 |
| **Total MUFA (g)** | 0.000001 | 0.526442 | 0.000001 | 0.000001 |
| **Total folate (µg)** | 0.000001 | 0.550481 | 0.000001 | 0.000001 |
| **Chloride (mg)** | -0.000001 | 0.512019 | 0.000001 | -0.000001 |
| **MUFA 18:1 (g)** | 0.000001 | 0.516827 | 0.000001 | 0.000001 |
| **Energy (kcal)** | 0.000001 | 0.620192 | 0.000000 | 0.000000 |
| **Tryptophan (mg)** | 0.000001 | 0.509615 | 0.000000 | 0.000000 |
| **Protein (g)** | -0.000001 | 0.512019 | 0.000000 | 0.000000 |
| | | **NW vs. OO** | | |
| **PUFA 18:4 (g)** | 0.166555 | 0.692935 | 0.115412 | 0.115412 |
| **PUFA 22:5 (g)** | -0.048660 | 0.542120 | 0.026380 | -0.026380 |
| **Chromium (mg)** | -0.026352 | 0.633152 | 0.016685 | -0.016685 |
| **MUFA 22:1 (g)** | -0.031161 | 0.521739 | 0.016258 | -0.016258 |
| **SFA 24:0 (g)** | -0.024488 | 0.520380 | 0.012743 | -0.012743 |
| **SFA 17:0 (g)** | -0.014660 | 0.607337 | 0.008903 | -0.008903 |
| **MUFA 14:1 (g)** | -0.014341 | 0.592391 | 0.008496 | -0.008496 |
| **SFA 15:0 (g)** | -0.015274 | 0.547554 | 0.008363 | -0.008363 |
| **SFA 22:0 (g)** | -0.013888 | 0.539402 | 0.007491 | -0.007491 |

| | | | | |
|---|---|---|---|---|
| **γ-Tocotrienol (mg)** | 0.012799 | 0.551630 | 0.007060 | 0.007060 |
| **α-Tocotrienol (mg)** | -0.011775 | 0.595109 | 0.007007 | -0.007007 |
| **β-Tocopherol (mg)** | -0.012084 | 0.565217 | 0.006830 | -0.006830 |
| **β-Tocotrienol (mg)** | 0.010209 | 0.555707 | 0.005673 | 0.005673 |
| **SFA 20:0 (g)** | -0.009438 | 0.573370 | 0.005412 | -0.005412 |
| **PUFA 20:5 (g)** | 0.007386 | 0.559783 | 0.004135 | 0.004135 |
| **PUFA 20:4 (g)** | -0.006304 | 0.592391 | 0.003735 | -0.003735 |
| **Thiamin (mg)** | 0.002490 | 0.641304 | 0.001597 | 0.001597 |
| **Galactose (g)** | -0.002855 | 0.524457 | 0.001497 | -0.001497 |
| **MUFA 20:1 (g)** | 0.002772 | 0.535326 | 0.001484 | 0.001484 |
| **PUFA 22:6 (g)** | 0.002247 | 0.581522 | 0.001307 | 0.001307 |
| **Pyridoxine (vitamin B6) (mg)** | -0.001663 | 0.665761 | 0.001107 | -0.001107 |
| **MUFA 16:1 (g)** | -0.001738 | 0.600543 | 0.001044 | -0.001044 |
| **SFA 10:0 (g)** | -0.001336 | 0.529891 | 0.000708 | -0.000708 |
| **Maltose (g)** | 0.001129 | 0.603261 | 0.000681 | 0.000681 |
| **SFA 4:0 (g)** | -0.001156 | 0.535326 | 0.000619 | -0.000619 |
| **Total isoflavones (mg)** | 0.001016 | 0.562500 | 0.000571 | 0.000571 |
| **Soluble fiber (g)** | -0.000948 | 0.516304 | 0.000489 | -0.000489 |
| **δ-Tocopherol (mg)** | 0.000660 | 0.630435 | 0.000416 | 0.000416 |
| **PUFA 18:3 (g)** | 0.000623 | 0.644022 | 0.000402 | 0.000402 |
| **SFA 6:0 (g)** | -0.000663 | 0.524457 | 0.000347 | -0.000347 |
| **Pantothenic acid (mg)** | -0.000544 | 0.614130 | 0.000334 | -0.000334 |
| **SFA 12:0 (g)** | 0.000515 | 0.502717 | 0.000259 | 0.000259 |
| **Crude fiber (g)** | -0.000355 | 0.627717 | 0.000223 | -0.000223 |
| **Manganese (mg)** | 0.000361 | 0.524457 | 0.000189 | 0.000189 |
| **Vitamin E (α-tocopherol) (mg)** | -0.000248 | 0.548913 | 0.000136 | -0.000136 |
| **Riboflavin (mg)** | 0.000172 | 0.521739 | 0.000090 | 0.000090 |
| **Zinc (mg)** | -0.000143 | 0.622283 | 0.000089 | -0.000089 |
| **Salt (g)** | -0.000119 | 0.551630 | 0.000066 | -0.000066 |
| **Trans fatty acids (g)** | 0.000121 | 0.539402 | 0.000065 | 0.000065 |
| **Vitamin D (μg)** | 0.000114 | 0.546196 | 0.000062 | 0.000062 |
| **Biotin (μg)** | -0.000090 | 0.673913 | 0.000060 | -0.000060 |
| **MUFA 18:1 (g)** | -0.000089 | 0.633152 | 0.000056 | -0.000056 |
| **Niacin (mg)** | -0.000089 | 0.584239 | 0.000052 | -0.000052 |
| **SFA 14:0 (g)** | -0.000102 | 0.510870 | 0.000052 | -0.000052 |
| **Total PUFA (g)** | 0.000084 | 0.586957 | 0.000049 | 0.000049 |
| **PUFA 18:2 (g)** | 0.000081 | 0.592391 | 0.000048 | 0.000048 |
| **Copper (mg)** | 0.000085 | 0.524457 | 0.000045 | 0.000045 |
| **Vitamin E (mg)** | -0.000076 | 0.527174 | 0.000040 | -0.000040 |
| **Fructose (g)** | -0.000066 | 0.578804 | 0.000038 | -0.000038 |
| **Total fiber (g)** | -0.000054 | 0.570652 | 0.000031 | -0.000031 |

| | | | | |
|---|---|---|---|---|
| **Glucose (g)** | -0.000048 | 0.576087 | 0.000027 | -0.000027 |
| **Vitamin E (IU)** | -0.000051 | 0.529891 | 0.000027 | -0.000027 |
| **Total MUFA (g)** | -0.000044 | 0.595109 | 0.000026 | -0.000026 |
| **Phytosterols (mg)** | -0.000036 | 0.694293 | 0.000025 | -0.000025 |
| **β-Sitosterol (mg)** | -0.000032 | 0.622283 | 0.000020 | -0.000020 |
| **Added sugar (g)** | 0.000033 | 0.544837 | 0.000018 | 0.000018 |
| **Protein (g)** | -0.000026 | 0.584239 | 0.000015 | -0.000015 |
| **Folic acid (µg)** | 0.000013 | 0.690217 | 0.000009 | 0.000009 |
| **Available carbohydrate (g)** | -0.000014 | 0.519022 | 0.000007 | -0.000007 |
| **Lactose (g)** | 0.000013 | 0.544837 | 0.000007 | 0.000007 |
| **Selenium (µg)** | 0.000013 | 0.543478 | 0.000007 | 0.000007 |
| **Theobromine (mg)** | -0.000010 | 0.563859 | 0.000005 | -0.000005 |
| **Total SFA (g)** | -0.000009 | 0.505435 | 0.000004 | -0.000004 |
| **Molybdenum (µg)** | -0.000007 | 0.580163 | 0.000004 | -0.000004 |
| **Choline (mg)** | -0.000007 | 0.633152 | 0.000004 | -0.000004 |
| **β-Cryptoxanthin (µg)** | -0.000006 | 0.573370 | 0.000004 | -0.000004 |
| **Food folate (µg)** | -0.000006 | 0.570652 | 0.000003 | -0.000003 |
| **Caffeine (mg)** | 0.000005 | 0.557065 | 0.000003 | 0.000003 |
| **Magnesium (mg)** | -0.000005 | 0.554348 | 0.000003 | -0.000003 |
| **Cholesterol (mg)** | -0.000004 | 0.567935 | 0.000002 | -0.000002 |
| **Vitamin D (IU)** | 0.000003 | 0.581522 | 0.000002 | 0.000002 |
| **Iron (mg)** | 0.000002 | 0.502717 | 0.000001 | 0.000001 |
| **Lutein and zeaxanthin (µg)** | -0.000002 | 0.755435 | 0.000001 | -0.000001 |
| **Cystine (mg)** | -0.000002 | 0.565217 | 0.000001 | -0.000001 |
| **Vitamin A (RE)** | -0.000002 | 0.714674 | 0.000001 | -0.000001 |
| **Carbohydrate (g)** | 0.000002 | 0.573370 | 0.000001 | 0.000001 |
| **Tryptophan (mg)** | -0.000001 | 0.529891 | 0.000001 | -0.000001 |

*Note: Regression analysis performed via Ridge regression, adjusted for gender. The top nutrients were selected as reaching a coefficients larger Q25 and an AUC >0.5 and were used in the rest of analyses. Abbreviations: AUC, area under the curve; DFE, dietary folate equivalents; MUFA, monounsaturated fatty acid; NW, normal weight; NW-A, normal weight with asthma; OO, overweight or obesity; OO-A, overweight or obesity with asthma; PUFA, polyunsaturated fatty acid; RE, retinol equivalents; SFA, saturated fatty acid.*

**Supplementary Table 3: Statistical summary of significant correlations between nutrient intake and pulmonary function parameters in NW-A**

| **Nutrients** | | **sRaw** | **TLC** | **VC** | **FRCpleth** | **LCI** | **FeNO** | **FEF 25-75%** | **FEV1/FVC** |
|---|---|---|---|---|---|---|---|---|---|
| **SFA 14:0 (g)** | Correlation | | | | **0.803** | | | | |
| | Significance | | | | 0.005 | | | | |
| **PUFA 18:3 (g)** | Correlation | | **0.815** | **0.709** | | | | | **-0.715** |
| | Significance | | 0.004 | 0.022 | | | | | 0.020 |
| **MUFA 20:1 (g)** | Correlation | | **0.743** | **0.828** | | | | | |
| | Significance | | 0.014 | 0.003 | | | | | |

| | | | | | | | | | |
|---|---|---|---|---|---|---|---|---|---|
| **SFA 20:0 (g)** | Correlation | | **0.785** | | | | | | |
| | Significance | | 0.007 | | | | | | |
| **Sucrose (g)** | Correlation | **0.823** | | | | | | | |
| | Significance | 0.003 | | | | | | | |
| **Available carbohydrate (g)** | Correlation | | | | | | | | **-0.808** |
| | Significance | | | | | | | | 0.005 |
| **Biotin (µg)** | Correlation | | | | | | **-0.686** | | |
| | Significance | | | | | | 0.007 | | |
| **Tryptophan (mg)** | Correlation | **0.842** | | | | | | **-0.607** | **-0.846** |
| | Significance | 0.002 | | | | | | 0.016 | 0.002 |
| **Niacin equivalents (mg)** | Correlation | **0.732** | | | | | | **-0.669** | **-0.764** |
| | Significance | 0.016 | | | | | | 0.006 | 0.010 |
| **Fluoride (µg)** | Correlation | **0.809** | | | | | | | **-0.655** |
| | Significance | 0.005 | | | | | | | 0.040 |
| **β-Sitosterol (mg)** | Correlation | **0.694** | | | | | | **-0.655** | **-0.809** |
| | Significance | 0.026 | | | | | | 0.008 | 0.005 |
| **Soluble fiber (g)** | Correlation | | | | | **-0.945** | | | |
| | Significance | | | | | 0.000 | | | |

*Note: Pearson's partial correlation performed on ranked data, controlling for sex. Strength and significance of associations are denoted by correlation coefficients, r (in bold), and corresponding two-sided p-values. Abbreviations: FEF 25-75%, forced mid-expiratory flow; FeNO, fractional exhaled nitric oxide; FEV1/FVC, forced expiratory volume in 1 second/forced vital capacity ratio; FRCpleth, functional residual capacity by plethysmography; LCI, lung clearance index; MUFA, monounsaturated fatty acid; NW-A, normal weight with asthma; PUFA, polyunsaturated fatty acid; SFA, saturated fatty acid; sRaw, specific airway resistance; TLC, total lung capacity; VC, vital capacity*

**Supplementary Table 4: Statistical summary of significant correlations between nutrient intake and pulmonary function parameters in OO-A**

| **Nutrients** | | **VC** | **TLC** | **IC** | **FVC** | **FEV1** | **FeNO** | **LCI** | **FEF 25-75%** | **FEV1/FVC** |
|---|---|---|---|---|---|---|---|---|---|---|
| **SFA 22:0 (g)** | Correlation | **0.752** | **0.694** | **0.631** | **0.738** | **0.492** | | **-0.575** | | |
| | Significance | 0.000 | 0.000 | 0.002 | 0.000 | 0.013 | | 0.005 | | |
| **MUFA 18:1 (g)** | Correlation | **0.615** | **0.584** | **0.699** | **0.667** | **0.490** | | **-0.526** | | |
| | Significance | 0.003 | 0.005 | 0.000 | 0.000 | 0.013 | | 0.012 | | |
| **PUFA 18:3 (g)** | Correlation | **0.686** | **0.652** | **0.497** | **0.668** | **0.512** | | **-0.501** | | |
| | Significance | 0.001 | 0.001 | 0.022 | 0.000 | 0.009 | | 0.018 | | |
| **PUFA 18:2 (g)** | Correlation | **0.637** | **0.616** | **0.586** | **0.604** | | | | | |
| | Significance | 0.002 | 0.003 | 0.005 | 0.001 | | | | | |
| **Total PUFA (g)** | Correlation | **0.581** | **0.559** | **0.492** | **0.568** | | | | | |
| | Significance | 0.006 | 0.008 | 0.024 | 0.003 | | | | | |
| **Total MUFA (g)** | Correlation | **0.493** | **0.514** | **0.583** | **0.570** | **0.415** | | **-0.459** | | |
| | Significance | 0.023 | 0.017 | 0.006 | 0.003 | 0.039 | | 0.032 | | |
| **β-Tocopherol (mg)** | Correlation | **0.715** | **0.702** | **0.716** | **0.642** | | | | | **-0.443** |
| | Significance | 0.000 | 0.000 | 0.000 | 0.001 | | | | | 0.034 |
| **Niacin equivalents (mg)** | Correlation | **0.746** | **0.616** | **0.659** | **0.564** | | | | | |
| | Significance | 0.005 | 0.003 | 0.001 | 0.003 | | | | | |

| | | | | | | | | | | |
|---|---|---|---|---|---|---|---|---|---|---|
| **Tryptophan (mg)** | Correlation | **0.659** | **0.537** | **0.644** | **0.500** | | | | | |
| | Significance | 0.001 | 0.012 | 0.002 | 0.011 | | | | | |
| **SFA 18:0 (g)** | Correlation | **0.576** | **0.575** | **0.570** | **0.521** | | | | | |
| | Significance | 0.006 | 0.006 | 0.007 | 0.008 | | | | | |
| **Niacin (mg)** | Correlation | **0.539** | **0.487** | **0.529** | **0.515** | | | | | |
| | Significance | 0.012 | 0.025 | 0.014 | 0.008 | | | | | |
| **δ-Tocopherol (mg)** | Correlation | **0.587** | **0.586** | **0.523** | **0.448** | | | | | |
| | Significance | 0.005 | 0.005 | 0.015 | 0.025 | | | | | |
| **γ-Tocopherol (mg)** | Correlation | **0.623** | **0.643** | **0.552** | **0.512** | | | | | **-0.491** |
| | Significance | 0.003 | 0.002 | 0.009 | 0.009 | | | | | 0.017 |
| **SFA 24:0 (g)** | Correlation | **0.603** | **0.582** | **0.464** | **0.494** | | | | | **-0.440** |
| | Significance | 0.004 | 0.006 | 0.034 | 0.012 | | | | | 0.036 |
| **Total folate (µg)** | Correlation | **0.504** | | **0.573** | **0.504** | | | | | |
| | Significance | 0.020 | | 0.007 | 0.010 | | | | | |
| **Thiamin (mg)** | Correlation | | | **0.544** | **0.517** | **0.420** | | | | |
| | Significance | | | 0.011 | 0.008 | 0.037 | | | | |
| **Total fiber (g)** | Correlation | **0.501** | | **0.595** | **0.505** | **0.429** | | | | |
| | Significance | 0.021 | | 0.004 | 0.010 | 0.032 | | | | |
| **α-Tocopherol (mg)** | Correlation | **0.490** | | **0.571** | **0.583** | **0.436** | | **-0.474** | | |
| | Significance | 0.024 | | 0.007 | 0.002 | 0.030 | | 0.026 | | |
| **Carbohydrate (g)** | Correlation | **0.477** | | **0.475** | **0.452** | | | | | **-0.532** |
| | Significance | 0.029 | | 0.029 | 0.023 | | | | | 0.009 |
| **Added sugar (g)** | Correlation | | | | | | **0.595** | | | |
| | Significance | | | | | | 0.003 | | | |
| **Protein (g)** | Correlation | | | | | | | | **-0.512** | **-0.615** |
| | Significance | | | | | | | | 0.009 | 0.002 |
| **Cholesterol (g)** | Correlation | | | | | **-0.425** | | | **-0.510** | **-0.461** |
| | Significance | | | | | 0.034 | | | 0.009 | 0.027 |
| **DII score** | Correlation | **-0.448** | | **-0.565** | **-0.491** | **-0.445** | | | | |
| | Significance | 0.042 | | 0.008 | 0.013 | 0.026 | | | | |

*Note: Pearson's partial correlation performed on ranked data, controlling for sex. Strength and significance of associations are denoted by correlation coefficients, r (in bold), and corresponding two-sided p-values. Abbreviations: DII, dietary inflammatory index; FEF 25-75%, forced mid-expiratory flow; FeNO, fractional exhaled nitric oxide; FEV1, forced expiratory volume in 1 second; FEV1/FVC, forced expiratory volume in 1 second/forced vital capacity ratio; FVC, forced vital capacity; IC, inspiratory capacity; LCI, lung clearance index; MUFA, monounsaturated fatty acid; OO-A, overweight or obesity with asthma; PUFA, polyunsaturated fatty acid; SFA, saturated fatty acid; TLC, total lung capacity; VC, vital capacity.*

## Supplementary Table 5: Statistical summary of significant correlations between nutrient intake and clinical parameters in NW-A

| **Nutrients** | | **TSH** | **RBC** | **Serum Vitamin D** | **Eosinophils** | **Platelets** | **Eczema** | **CO2** | **Heart Rate** | **C-peptide** |
|---|---|---|---|---|---|---|---|---|---|---|
| **DII score** | Correlation | | | **-0.652** | | | | | | **0.613** |
| | Significance | | | 0.008 | | | | | | 0.020 |
| **Iodine (µg)** | Correlation | | | | | **0.651** | **0.612** | | | |
| | Significance | | | | | 0.009 | 0.015 | | | |
| | Correlation | | **0.691** | | | | | | | |

| | | | | | | | | | | |
|---|---|---|---|---|---|---|---|---|---|---|
| **SFA 12:0 (g)** | Significance | | 0.004 | | | | | | | |
| **Glucose (g)** | Correlation | | | | | | | | | **-0.700** |
| | Significance | | | | | | | | | 0.005 |
| **Fructose (g)** | Correlation | | | **0.643** | | | | | | **-0.754** |
| | Significance | | | 0.010 | | | | | | 0.002 |
| **Soluble fiber (g)** | Correlation | **0.632** | | **0.560** | | | | | | **-0.795** |
| | Significance | 0.011 | | 0.03 | | | | | | 0.001 |
| **Insoluble fiber (g)** | Correlation | **0.590** | | | | | | | | **-0.700** |
| | Significance | 0.021 | | | | | | | | 0.005 |
| **Food folate (µg)** | Correlation | | | **0.651** | | | | | | |
| | Significance | | | 0.009 | | | | | | |
| **Crude fiber (g)** | Correlation | | | | | | | **-0.811** | | |
| | Significance | | | | | | | 0.004 | | |
| **Total fiber (g)** | Correlation | | | | | **0.582** | **-0.684** | | | **-0.672** |
| | Significance | | | | | 0.023 | 0.005 | | | 0.008 |
| **Folic acid (µg)** | Correlation | | | | **0.676** | | | | **-0.648** | **-0.539** |
| | Significance | | | | 0.006 | | | | 0.009 | 0.046 |
| **MUFA 14:1 (g)** | Correlation | | | | | | | | **-0.762** | |
| | Significance | | | | | | | | 0.001 | |
| **Lactose (g)** | Correlation | | | | **-0.664** | | | | | |
| | Significance | | | | 0.007 | | | | | |

*Note: Pearson's partial correlation was performed on ranked data, controlling for sex. Strength and significance of associations are denoted by correlation coefficients, r (in bold), and corresponding two-sided p-values. Abbreviations: NW-A: normal weight with asthma; DII, diet inflammatory index; SFA, staurated fatty acids; MUFA: monounsaturated fatty acids.*

## Supplementary Table 6: Statistical summary of significant correlations between nutrient intake and clinical parameters in OO-A

| **Nutrients** | | **Albumin** | **RBC** | **Serum vitamin D** | **HDL cholesterol** | **C-peptide** | **Allergies** | **Rhinitis** | **Heart rate** |
|---|---|---|---|---|---|---|---|---|---|
| **Phytosterols (mg)** | Correlation | | **0.405** | **0.449** | | **-0.551** | | | |
| | Significance | | 0.045 | 0.031 | | 0.006 | | | |
| **Iron (mg)** | Correlation | **0.646** | **0.403** | | | | | | **-0.480** |
| | Significance | 0.000 | 0.046 | | | | | | 0.015 |
| **Available carbohydrate (g)** | Correlation | | | **0.540** | | | | | |
| | Significance | | | 0.008 | | | | | |
| **MUFA 16:1 (g)** | Correlation | | | | | | | | **-0.564** |
| | Significance | | | | | | | | 0.003 |
| **Caffeine (mg)** | Correlation | | **0.560** | | | | | **-0.699** | **-0.405** |
| | Significance | | 0.004 | | | | | <0.001 | 0.044 |
| **Theobromine (mg)** | Correlation | | | | | **-0.476** | | **-0.638** | |
| | Significance | | | | | 0.022 | | <0.001 | |
| **Lactose (g)** | Correlation | | | | **0.540** | | | | |
| | Significance | | | | 0.009 | | | | |
| **Trans Fat (g)** | Correlation | | | | | | **-0.529** | | |
| | Significance | | | | | | 0.007 | | |

*Note: Pearson's partial correlation was performed on ranked data, controlling for sex. Strength and significance of associations are denoted by correlation coefficients, r (in bold), and corresponding two-sided p-values.*

*Abbreviations: HDL: high density lipoprotein; MUFA: monounsaturated fatty acid; OO-A: overweight or obesity with asthma; RBC, red blood cells.*

**Supplementary Table 7: Statistical summary of cytokines showing significant variation across the four study groups**

| Cytokines | NW-A | OO-A | NW | OO | p-value | Pairwise comparison (p-value) |
|---|---|---|---|---|---|---|
| **IL-5 (pg/mL)** | 12.5 (5.00-33.3) | 14.0 (8.00-27.5) | 5.00 (2.00-16.8) | 16.0 (7.00-34.8) | 0.004 | OO-A vs. NW (p=0.011)<br>NW vs. OO (p=0.005) |
| **IL-33 (pg/mL)** | 17.0 (4.50-57.0) | 17.5 (5.00-49.0) | 2.00 (0.00-14.3) | 13.0 (8.00-44.0) | 0.004 | NW vs. OO (p=0.026) |
| **IL-13 (pg/mL)** | 18.0 (8.50-46.5) | 12.50 (0.75-31.3) | 2.00 (0.00-7.00) | 7.00 (0.00-27.3) | 0.033 | NW-A vs. NW (p=0.014) |
| **TNF-α (pg/mL)** | 10.5 (5.25-23.5) | 12.0 (7.75-15.3) | 6.50 (4.00-10.0) | 10.5 (5.50-15.0) | 0.032 | ns |
| **Leptin (pg/mL)** | 930.5 (505.3-4111) | 7114(4598 - 11193) | 976 (485 – 3487) | 6081 (1316-8970) | <0.001 | NW-A vs. OO-A (p=0.002)<br>NW-A vs. OO (p=0.003)<br>OO-A vs. NW (p<0.001)<br>NW vs. OO (p<0.001) |

*Note: Blood cytokine levels are presented as median (IQR). Group comparisons were performed via the Kruskal-Wallis test, followed by Bonferroni-corrected Dunn's post hoc test. Abbreviations: IL, interleukin; ns, not significant; NW, normal weight; NW-A, normal weight with asthma; OO, overweight or obesity; OO-A, overweight or obesity with asthma; TNF, tumor necrosis factor.*

**Supplementary Table 8: Statistical summary of significant correlations between nutrient intake and plasma cytokine levels in NW-A**

| Nutrients | | Leptin | TNF-α | IL-33 | IL-2 | IL-5 | IFN-γ | IL-17A | IL-13 | IL-10 |
|---|---|---|---|---|---|---|---|---|---|---|
| **Vitamin D (µg)** | Correlation | | | | | **0.631** | **0.670** | **0.572** | **0.619** | |
| | Significance | | | | | 0.012 | 0.006 | 0.026 | 0.014 | |
| **DII score** | Correlation | | | | **0.534** | | | | | |
| | Significance | | | | 0.040 | | | | | |
| **Iodine (µg)** | Correlation | | | | | | | | | **0.586** |
| | Significance | | | | | | | | | 0.022 |
| **Isoflavones (mg)** | Correlation | **-0.593** | | | | | | | | |
| | Significance | 0.020 | | | | | | | | |
| **Pantothenic acid (mg)** | Correlation | **-0.625** | | | | | | | | |
| | Significance | 0.013 | | | | | | | | |
| **SFA 14:0 (g)** | Correlation | | | | | | | | **-0.517** | |
| | Significance | | | | | | | | 0.049 | |
| **Carbohydrate (g)** | Correlation | **-0.519** | | | | | | | | |
| | Significance | 0.047 | | | | | | | | |
| **Folate (DFE) (µg)** | Correlation | | | | | | | | | **-0.535** |
| | Significance | | | | | | | | | 0.040 |
| **Total fiber (g)** | Correlation | | | | | | | | | **-0.558** |
| | Significance | | | | | | | | | 0.031 |
| **Thiamin (mg)** | Correlation | | | | | | | | | **-0.535** |
| | Significance | | | | | | | | | 0.040 |
| **PUFA 18:3 (g)** | Correlation | | | | | | | | | **-0.662** |
| | Significance | | | | | | | | | 0.007 |
| **MUFA 16:1 (g)** | Correlation | | | | | | | | | **-0.516** |

| | | | | | | | | | | |
|---|---|---|---|---|---|---|---|---|---|---|
| | Significance | | | | | | | | | 0.049 |
| **SFA 4:0 (g)** | Correlation | | | | | | | | **-0.608** | |
| | Significance | | | | | | | | 0.016 | |
| **Total MUFA (g)** | Correlation | | | | | | | | | **-0.537** |
| | Significance | | | | | | | | | 0.039 |
| **SFA 18:0 (g)** | Correlation | | | | | | | | **-0.547** | **-0.696** |
| | Significance | | | | | | | | 0.035 | 0.004 |
| **SFA 16:0 (g)** | Correlation | | | | | | | | **-0.613** | **-0.783** |
| | Significance | | | | | | | | 0.015 | 0.001 |
| **MUFA 18:1 (g)** | Correlation | | | | | | | **-0.529** | **-0.539** | **-0.744** |
| | Significance | | | | | | | 0.043 | 0.038 | 0.001 |
| **Vitamin E (mg)** | Correlation | | **-0.524** | | | | | | | |
| | Significance | | 0.045 | | | | | | | |
| **SFA 8:0 (g)** | Correlation | | | | | | | | **-0.532** | |
| | Significance | | | | | | | | 0.041 | |
| **Sucrose (g)** | Correlation | | | | **-0.583** | | | **-0.607** | | |
| | Significance | | | | 0.023 | | | 0.016 | | |
| **Lactose (g)** | Correlation | | | | **-0.529** | | | **-0.600** | | |
| | Significance | | | | 0.043 | | | 0.018 | | |
| **SFA 15:0 (g)** | Correlation | | | | | | | **-0.576** | | |
| | Significance | | | | | | | 0.025 | | |
| **MUFA 14:1 (g)** | Correlation | | | | | | **-0.639** | **-0.641** | | |
| | Significance | | | | | | 0.010 | 0.010 | | |
| **α-Tocopherol (mg)** | Correlation | | | | | | | | **-0.598** | **-0.623** |
| | Significance | | | | | | | | 0.019 | 0.013 |
| **MUFA 22:1 (g)** | Correlation | | **-0.595** | | | | **-0.529** | **-0.604** | **-0.554** | **-0.602** |
| | Significance | | 0.019 | | | | 0.042 | 0.017 | 0.032 | 0.017 |
| **SFA 12:0 (g)** | Correlation | | | | | **-0.592** | **-0.623** | **-0.563** | **-0.612** | **-0.560** |
| | Significance | | | | | 0.020 | 0.013 | 0.029 | 0.015 | 0.030 |
| **MUFA 20:1 (g)** | Correlation | | | | | | **-0.590** | **-0.542** | **-0.562** | **-0.730** |
| | Significance | | | | | | 0.020 | 0.037 | 0.029 | 0.002 |
| **SFA 20:0 (g)** | Correlation | | | **-0.528** | | **-0.525** | **-0.584** | **-0.631** | **-0.613** | **-0.758** |
| | Significance | | | 0.043 | | 0.044 | 0.022 | 0.012 | 0.015 | 0.001 |
| **PUFA 18:2 (g)** | Correlation | | **-0.578** | **-0.584** | **-0.551** | | **-0.554** | **-0.640** | **-0.550** | **-0.790** |
| | Significance | | 0.024 | 0.022 | 0.033 | | 0.032 | 0.010 | 0.034 | 0.000 |

*Note: Pearson's partial correlation was performed on ranked data, controlling for sex. Strength and significance of associations are denoted by correlation coefficients, r (in bold), and corresponding two-sided p-values. Abbreviations: DFE, dietary folate equivalents; DII, dietary inflammatory index; IFN, interferon; IL, interleukin; MUFA, monounsaturated fatty acid; NW-A, normal weight with asthma; PUFA, polyunsaturated fatty acid; SFA, saturated fatty acid; TNF, tumor necrosis factor.*

## Supplementary Table 9: Statistical summary of significant correlations between nutrient intake and plasma cytokine levels in OO-A

| **Nutrients** | | **IL-22** | **IFN-γ** | **TNF-α** | **IL-13** | **IL-5** | **IL-10** | **IL-17A** | **IL-2** |
|---|---|---|---|---|---|---|---|---|---|
| **Fructose (g)** | Correlation | **0.404** | | | | | | | |
| | Significance | 0.045 | | | | | | | |
| **Energy (kcal)** | Correlation | | | | **-0.405** | | | | |
| | Significance | | | | 0.045 | | | | |
| **Galactose (g)** | Correlation | | | | **-0.410** | | | | |
| | Significance | | | | 0.042 | | | | |
| **Vitamin E (mg)** | Correlation | | | | **-0.536** | | | | |
| | Significance | | | | 0.006 | | | | |

| | | | | | | | | | |
|---|---|---|---|---|---|---|---|---|---|
| **Total fiber (g)** | Correlation | | | | | **-0.467** | | | |
| | Significance | | | | | 0.019 | | | |
| **SFA 17:0 (g)** | Correlation | | | | | | **-0.413** | | |
| | Significance | | | | | | 0.040 | | |
| **SFA 18:0 (g)** | Correlation | | | | | | **-0.406** | | **-0.418** |
| | Significance | | | | | | 0.044 | | 0.037 |
| **Zinc (mg)** | Correlation | | | | | | **-0.406** | | |
| | Significance | | | | | | 0.044 | | |
| **Pantothenic acid (mg)** | Correlation | | | | | | **-0.410** | | |
| | Significance | | | | | | 0.042 | | |
| **MUFA 18:1 (g)** | Correlation | | | | | | | | **-0.409** |
| | Significance | | | | | | | | 0.042 |
| **Total MUFA (g)** | Correlation | | | | | | **-0.406** | | **-0.428** |
| | Significance | | | | | | 0.044 | | 0.033 |
| **Salt (g)** | Correlation | | | **-0.463** | | | | | **-0.430** |
| | Significance | | | 0.020 | | | | | 0.032 |
| **Thiamin (mg)** | Correlation | | | | | **-0.430** | **-0.440** | **-0.419** | **-0.433** |
| | Significance | | | | | 0.032 | 0.028 | 0.037 | 0.030 |
| **Trans fatty acids (g)** | Correlation | | | | **-0.497** | **-0.439** | **-0.470** | | **-0.403** |
| | Significance | | | | 0.012 | 0.028 | 0.018 | | 0.046 |
| **Iron (mg)** | Correlation | | | | | | | **-0.430** | **-0.419** |
| | Significance | | | | | | | 0.032 | 0.037 |
| **Folate (DFE) (µg)** | Correlation | | | | | | | | **-0.436** |
| | Significance | | | | | | | | 0.029 |
| **Total folate (µg)** | Correlation | | | | | | | **-0.413** | **-0.482** |
| | Significance | | | | | | | 0.040 | 0.015 |
| **SFA 14:0 (g)** | Correlation | | | | | | | | **-0.493** |
| | Significance | | | | | | | | 0.012 |
| **SFA 4:0 (g)** | Correlation | | | **-0.412** | | | | **-0.474** | **-0.547** |
| | Significance | | | 0.041 | | | | 0.017 | 0.005 |
| **Folic acid (µg)** | Correlation | | **-0.428** | | | | | **-0.549** | **-0.568** |
| | Significance | | 0.033 | | | | | 0.004 | 0.003 |
| **Lactose (g)** | Correlation | | **-0.418** | **-0.477** | | | | **-0.520** | **-0.581** |
| | Significance | | 0.038 | 0.016 | | | | 0.008 | 0.002 |
| **β-Sitosterol (mg)** | Correlation | | **-0.508** | | | **-0.473** | **-0.452** | **-0.516** | **-0.495** |
| | Significance | | 0.010 | | | 0.017 | 0.023 | 0.008 | 0.012 |
| **Manganese (mg)** | Correlation | | **-0.435** | | **-0.508** | **-0.587** | **-0.562** | **-0.514** | **-0.510** |
| | Significance | | 0.030 | | 0.010 | 0.002 | 0.003 | 0.009 | 0.009 |

*Note: Pearson's partial correlation was performed on ranked data, controlling for sex. Strength and significance of associations are denoted by correlation coefficients, r (in bold), and corresponding two-sided p-values. Abbreviations: DFE, dietary folate equivalents; IFN, interferon; IL, interleukin; MUFA, monounsaturated fatty acid; OO-A, overweight or obesity with asthma; SFA, saturated fatty acid; TNF, tumor necrosis factor.*

## 2 Supplementary Figures

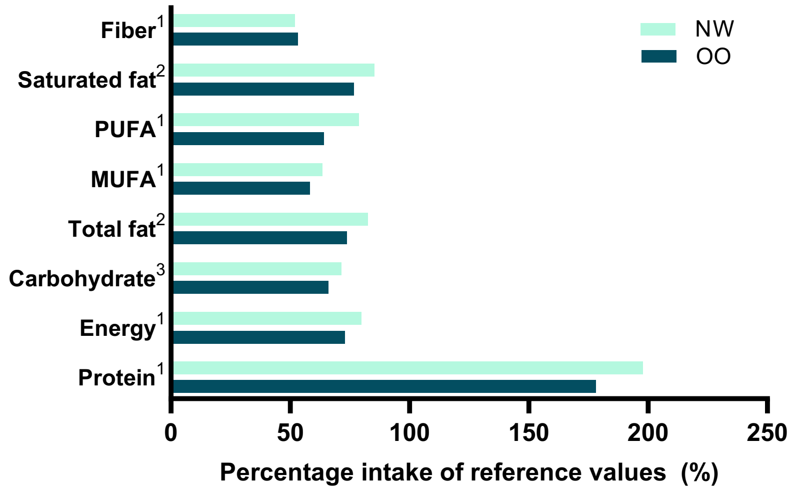


**Supplementary Figure 1. Quantitative dietary analysis of the NW and the OO groups**

Bar chart showing energy, macronutrient, and fiber intake in NW and OO compared to the UK Government Dietary Recommendations. Nutrient reference values represent either [1]average, [2]maximum, or [3]minimum recommended intake. Groups were compared via the Mann–Whitney U test or t-test. Abbreviations: MUFA, monounsaturated fatty acids; NW, normal weight; OO, overweight or obesity; PUFA, polyunsaturated fatty acids
